# Navigating oxidative stress in oral bone regeneration: mechanisms and reactive oxygen species-regulating biomaterial strategies

**DOI:** 10.1093/rb/rbaf091

**Published:** 2025-09-01

**Authors:** Lingling Liang, Xiaowen Li, Hao Liang, Jinzheng Zhang, Qinglan Lu, Guangqi Zhou, Jiajing Tang, Xiaojie Li

**Affiliations:** College & Hospital of Stomatology, Guangxi Medical University, Guangxi Key Laboratory of Oral and Maxillofacial Rehabilitation and Reconstruction, Nanning 530021, PR China; College & Hospital of Stomatology, Guangxi Medical University, Guangxi Key Laboratory of Oral and Maxillofacial Rehabilitation and Reconstruction, Nanning 530021, PR China; College & Hospital of Stomatology, Guangxi Medical University, Guangxi Key Laboratory of Oral and Maxillofacial Rehabilitation and Reconstruction, Nanning 530021, PR China; Research Center for Nano-Biomaterials, Analytical and Testing Center, Sichuan University, Chengdu 610064, PR China; College & Hospital of Stomatology, Guangxi Medical University, Guangxi Key Laboratory of Oral and Maxillofacial Rehabilitation and Reconstruction, Nanning 530021, PR China; College & Hospital of Stomatology, Guangxi Medical University, Guangxi Key Laboratory of Oral and Maxillofacial Rehabilitation and Reconstruction, Nanning 530021, PR China; College & Hospital of Stomatology, Guangxi Medical University, Guangxi Key Laboratory of Oral and Maxillofacial Rehabilitation and Reconstruction, Nanning 530021, PR China; College & Hospital of Stomatology, Guangxi Medical University, Guangxi Key Laboratory of Oral and Maxillofacial Rehabilitation and Reconstruction, Nanning 530021, PR China

**Keywords:** oral bone, bone defect, bone regeneration, oxidative stress, ROS-regulating biomaterials

## Abstract

‘Oral bone’ primarily refers to the bones within the mouth, specifically the jawbones and the alveolar bone that supports teeth. Oral bone tissue defects are commonly caused by trauma, inflammation and surgical excision and their repair represents one of the core challenges in the field of oral medicine. The use of functional biomaterials for tissue regeneration has become a research focus in the field of damaged tissue treatment. However, following the implantation of biomaterials, the immune response induces the generation of reactive oxygen species (ROS) and the open and susceptible environment of oral bone predisposes it to redox imbalance, resulting in ROS accumulation and compromised repair. In response to this challenge, ROS-regulating biomaterials have developed into an effective platform for restoring redox balance. Despite this progress, current research lacks a systematic framework for the mechanism and design of biomaterials specifically addressing the special metabolism of oral bone. This review focuses on the physiological and pathological characteristics of oral bone, explores the interaction mechanisms between the oxidative stress and oral bone defects and provides a functional classification of regulation mechanisms. In addition, this review provides several corresponding suggestions for the development of targeted biomaterials according to the problems of existing ROS-regulating materials applied in oral bone repair.

## Graphical abstract

**Figure:**
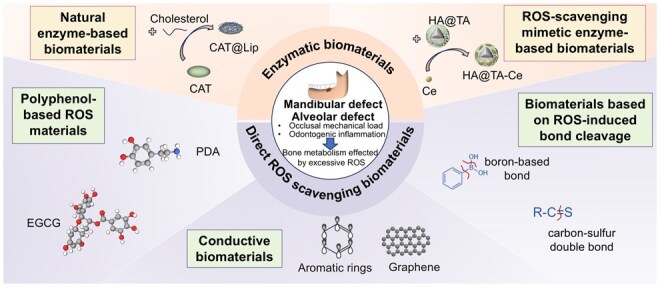

## Introduction

‘Oral bone’ primarily refers to the bones within the mouth, specifically the jawbones (maxilla and mandible) and the alveolar bone that supports teeth. Oral bone defects, primarily caused by trauma, inflammation and surgical resection, which not only lead to chewing, speech and respiratory dysfunction, but also lead to aesthetic and mental health problems [1]. It has become one of the core challenges in the field of oral medicine. Most bone defects could not heal naturally or only less than 10% of new bone formed without therapeutic intervention, which defined critical-sized bone defects [2, 3]. Although autologous bone transplantation is generally considered the ‘gold standard’ for bone repair, it has problems such as limited donor tissue sources and donor site complications [4]. With advancements in materials science, multifunctional biomaterials have emerged as a promising alternative therapeutic strategy [5].

Upon implantation of biomaterials into the body, immune cells, such as neutrophils and macrophages, generate reactive oxygen species (ROS) primarily through the nicotinamide adenine dinucleotide phosphate hydrogen oxidase (NOX) pathway and the mitochondrial respiratory chain [6, 7]. However, ROS production is not limited to immune cells, other types involved in bone repair and tissue regeneration, including fibroblasts, osteoblasts (OBs) and endothelial cells, also express NOX enzymes and can contribute to ROS accumulation via mitochondrial electron leakage [8]. This broader cellular contribution plays a critical role in modulating the redox environment at the implantation site [9]. In the early stages, low concentrations of ROS can facilitate tissue regeneration by eliminating pathogens and activating pro-repair signaling pathways [10]. However, if ROS production becomes excessive and sustained, it not only leads to the oxidation and degradation of the biomaterial surface (e.g. ester bond cleavage in polyester materials and the release of metal ions interacting with anions) but also damages surrounding cellular proteins and DNA, inhibiting the osteogenic differentiation of mesenchymal stem cells (MSCs) and inducing apoptosis [11, 12]. ROS can attack chemical bonds of biomaterials and lead to the breakdown of the material’s structure and potentially release harmful byproducts. For instance, metal ions released from the biomaterial can interact with anions in the surrounding environment, further contributing to degradation and potential toxicity [13, 14]. Compared to other bone tissues, oral and maxillofacial bones are subjected to long-term, high mechanical loads from chewing, which significantly enhances their cellular metabolism and bone remodeling activities, leading to a high turnover rate [15]. In this process, the synergistic action of OBs and osteoclasts (OCs) maintains bone homeostasis through a dynamic balance between bone formation and resorption [16, 17]. The active mitochondrial respiratory chain in OBs, along with the activation of OC oxidases, contributes to elevated ROS levels [18, 19]. Meanwhile, the body strives to maintain a delicate redox balance through antioxidant enzyme systems—such as superoxide dismutase (SOD) and glutathione peroxidase (GPx)—as well as oxidative repair mechanisms (e.g. thioredoxin) [20]. However, when factors like trauma, infection or surgery induce persistent inflammation, this redox balance is disrupted, leading to the accumulation of excessive ROS. Given the distinctive anatomical, microbial and mechanical environment of oral bone, the redox balance is particularly sensitive and dynamically regulated. Therefore, researchers are committed to integrating ROS regulatory properties into biomaterials for oral bone repair, hoping to restore redox balance by regulating ROS and thus promote bone repair. Specific types of antioxidant materials, such as microparticles/nanoparticles cerium dioxide (CeO_2_), have been successfully utilized to scavenge and prevent the generation of excessive ROS in tissue engineering applications [21]. However, existing studies vary widely in design, efficacy and applicability, often lacking spatiotemporal control, pathology-specific tuning or consideration of oral-specific challenges.

Despite the growing interest in redox-responsive materials, there remains a lack of comprehensive reviews that specifically focus on oral bone. Existing literature often overlooks the dual physiological and pathological roles of ROS in oral bone environments, as well as the unique challenges posed by the oral cavity’s microbial and inflammatory conditions. Furthermore, current reviews rarely offer a systematic classification of the mechanistic principles and design strategies employed in ROS-regulating biomaterials, nor do they sufficiently address the unmet challenges and design gaps in this rapidly evolving field. Therefore, this review is timely and necessary—it aims to clarify the complex interactions between ROS and oral bone pathophysiology, functionally categorize existing biomaterial strategies based on their ROS regulation mechanisms, and propose innovative frameworks for the design of disease-adaptive, phased and multithreshold biomaterials tailored to oral bone repair. This review conducted a literature search using the PubMed and Web of Science databases, as well as Google Scholar, with no restriction on publication year. The search was based on the following key terms (‘oxidative stress’ OR ‘reactive oxygen species’ OR ‘ROS’ OR ‘redox’ OR ‘antioxidant’) AND (‘bone defect’ OR ‘bone regeneration’) AND (‘oral bone’ OR ‘jawbone’ OR ‘maxilla’ OR ‘mandible’ OR ‘alveolar bone’). Only laboratory-based studies, clinical trials and review articles directly related to oral bone applications were included. This review is oriented to the clinical needs, focusing on the specific pathological characteristics of oral bone, filling gaps in the theoretical mechanism, material design and translational application of ROS-regulating biomaterials and providing innovative ideas and solution for overcoming critical oral bone defect repair challenges.

## Unique patterns of bone formation and resorption in the oral bone

Oral bone is a complex dynamic tissue undergoing continuous formation by OBs and resorption by OCs [18]. During bone reconstruction, OCs remove old or damaged bone tissue and OBs rebuild new bone. Osteocytes, located within the mineralized bone matrix, respond to microdamage and mechanical stress by recruiting OBs and promoting bone formation through the activation of the canonical wingless-related integration site (Wnt)/*β*-catenin signaling pathway [22]. This regulatory mechanism enables oral bone tissue has the ability to adapt to the constantly changing microenvironment surrounding it.

**Figure 1.**
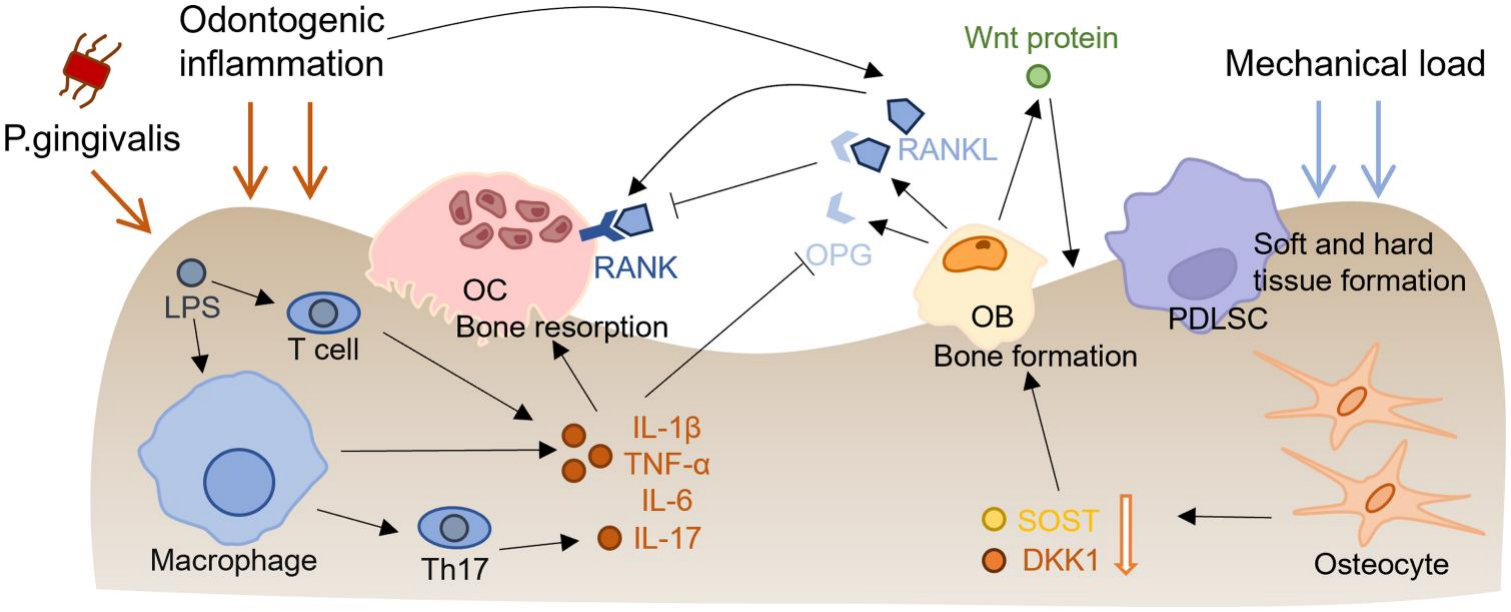
Mechanisms of metabolic turnover in the oral jaw and alveolar bone. Metabolic turnover in the oral jaw and alveolar bone is a dynamic process involving bone formation and resorption caused by infection, odontogenic inflammation and mechanical loading. → stimulatory modification. ⊥ inhibitory modification. ↓ filling with white means suppression. LPS: Lipopolysaccharide. PDLSC: Periodontal ligament stem cell.

Owing to constant occlusal stimulation and the prevalence of inflammatory conditions in the oral cavity, jawbones—particularly the maxilla and alveolar bone—exhibit higher metabolic and remodeling activity than other skeletal sites [23–25]. Structurally, while all bones consist of cortical bone surrounding trabecular bone, the maxilla contains up to 90% trabecular bone, in contrast to approximately 20% in long bones, resulting in a larger and more cellular endosteal surface area that supports rapid bone turnover [26]. Moreover, the alveolar bone endures mechanical loads approximately twice as high as those experienced by long bones [27]. Studies have shown that chewing activity under physiological conditions increases mandibular and alveolar bone density, as well as the width of the periodontal ligament, compared to the non-chewing side [28, 29]. The annual bone formation rates of the mandibular (36.9%) and maxillary (19.1%) alveolar bone in dogs were significantly higher—up to six times—than those of the femur (6.4%) [30]. In addition, occlusal loading during mastication has been shown to reduce sclerostin (SOST) and dickkopf-related protein 1 (DKK1) expression from osteocytes, thereby inhibiting bone resorption and promoting alveolar bone formation (Figure 1) [24, 31].

The oral cavity is an open environment that exposed to external factors such as oral bacteria. Current research shows that periodontal inflammation is a direct consequence of the inflammatory response with ROS generation [32]. This host response involves recruitment of inflammatory cells (e.g. macrophage, T cell) and production of cytokines (e.g. IL-1*β*, IL-17), which initiate OC activity and disturb the balance between protective and destructive processes (Figure 1) [33]. For example, Th17 cell, one of the major types of T cells, produces IL-17 that involved in the destruction of periodontal tissue after being attacked by porphyromonas gingivalis [34]. OBs express receptor activator of nuclear factor kappa-B ligand (RANKL) and secrete osteoprotegerin (OPG). OPG is a soluble receptor that binds RANKL and blocks its binding to the receptor activator of nuclear factor kappa-B (RANK), which results in suppression of OC activity [35]. However, immune cells produce IL-17 or other cytokines to stimulate OBs to express RANKL and inhibit the expression of OPG, leading to bone and connective tissue degradation [36, 37].

All of above reflect the role of mechanical loading and periodontal inflammation on oral bone cell metabolism. This section will detail the unique patterns of bone formation and resorption in oral bone and the role of ROS in this process.

### Physiological homeostasis of reactive oxygen species

**Figure 2.**
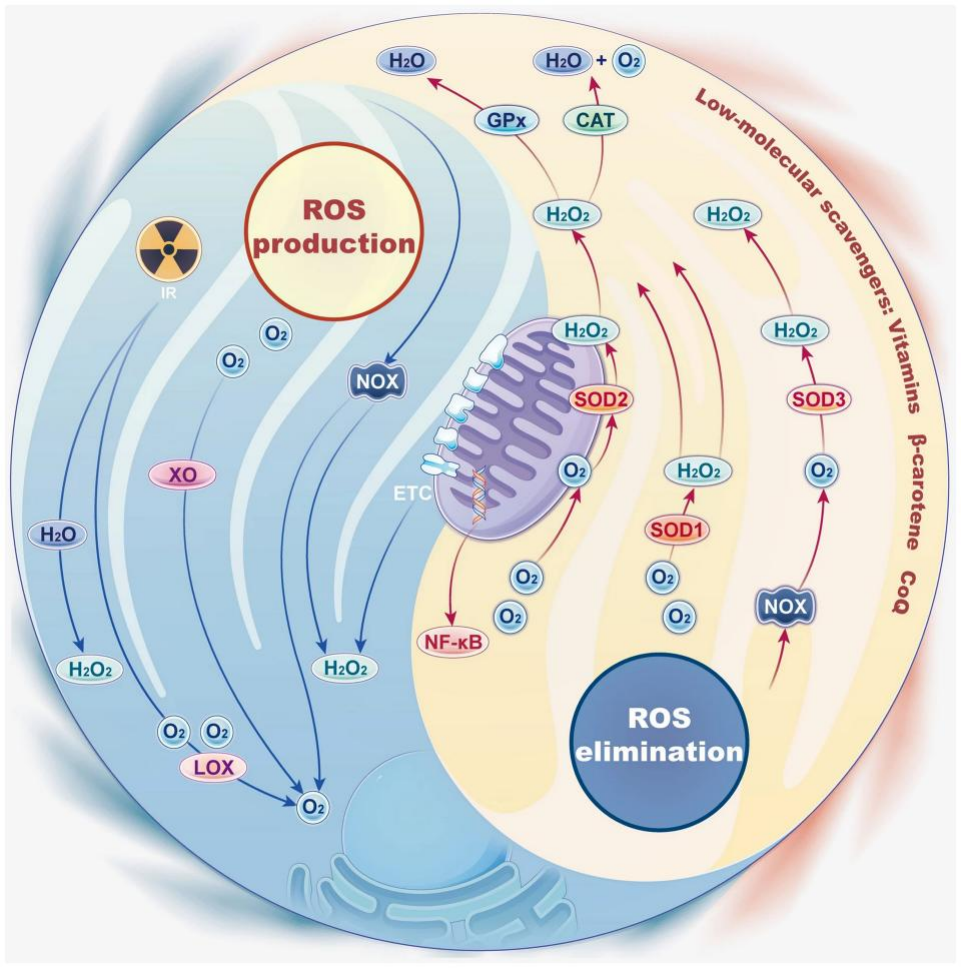
Production and elimination of ROS. ROS are mainly generated from the mitochondrial electron transport chain and also produced by several cellular enzymes, including NOX, XOR and LOX. To maintain balance, ROS is controlled with the help of antioxidant networks. The ROS elimination system comprises endogenous antioxidant enzymes and several low-molecular-weight eliminators, including SOD, CAT, GPx, vitamins, *β*-catenin, coenzyme Q, selenium and zinc. IR: Infrared spectroscopy. ETC: Electron transport chain. Reproduced with permission from Ref. [40]. Copyright 2023, BioMed Central.

ROS are involved in both the promotion and inhibition of bone formation and resorption, playing a significant role in the physiology and pathology of oral bones. Multiple studies reported a close relationship between ROS and bone turnover: bone turnover was controlled by ROS levels to inhibit OC differentiation [38]; ROS differentially regulated bone turnover in an age-specific manner [39]. Therefore, comprehending the role of ROS generation and elimination in the physiological processes of oral bone is essential, as it provides a foundation for the subsequent design and development of ROS-regulating biomaterials (Figure 2).

#### Reactive oxygen species generation

ROS are typically produced as by-products of biological reactions by the mitochondrial respiratory chain, while NOXs produce ROS as part of their primary function [9]. Physiological concentrations of ROS are essential for maintaining cellular redox homeostasis, influencing various signaling pathways and regulating cell proliferation. However, excessive ROS damages cellular components (e.g. DNA, lipids and proteins), leading to impaired cellular function and undesirable alterations.

The sources of ROS can be categorized in two ways. (1) ROS can be generated during metabolic processes in various systems of the body, especially normal physiological activities of the aerobic system and NOX activity [41]. Most ROS originate from the mitochondrial electron transport chain (METC), which generate approximately 90% of cellular ROS [42, 43]. During cellular oxidation, electrons are transferred through the METC and coupled with the generation of proton gradients across the inner mitochondrial membrane, but they may leak from complexes I, II and III to form superoxide ($O_{2}^{\cdot -}$) and disproportionate to hydrogen peroxide (H_2_O_2_) [44]. When mitochondrial membrane permeability is increased, ROS may be released into the cytoplasm, triggering inflammation or damaging biomolecules [45]. Cellular enzymes such as NOXs, xanthine oxidoreductases (XORs) and lipoxygenases (LOXs) produce ROS. NOXs contribute to a smaller amount compared to mitochondrial, but it is still significant, particularly in specific cellular contexts and signaling pathways [46]. NOXs generate ROS by catalyzing the transfer of electrons from nicotinamide adenine dinucleotide phosphate hydrogen (NADPH) to molecular oxygen [47]; XORs catalyze the production of uric acid from xanthine and xanthine oxidase (XO) could use oxygen (O_2_) to produce ROS [48]; LOXs catalyze the generation of ROS from fatty acids [49]. (2) Ionizing radiation (IR) is another source of ROS. Low linear energy transfer IR, including *γ*-rays and *x*-rays, generates ROS by exciting and ionizing water molecules (H_2_O). In addition, ROS can react with other H_2_O and O_2_ to produce more ROS through indirect effects [50].

#### Reactive oxygen species elimination

The antioxidant system of an organism is defined as the oxidative defense system, which consists of substances in lower concentrations than oxidative substrates that delay or prevent oxidation [51]. It is worth noting that antioxidant defense systems should maintain adequate ROS levels to perform essential functions, rather than significantly reducing them.

The human ROS-scavenging system can be categorized into enzymatic and non-enzymatic scavenging systems. (1) Among the enzymatic ROS-scavenging defenses are metalloenzymes that scavenge and inhibit free radicals, such as SOD, GPx, catalase (CAT) and metallothionein (MT). SOD catalyzes the dismutation of $O_{2}^{\cdot -}$ to O_2_ and H_2_O_2_; CAT converts H_2_O_2_ to H_2_O and O_2_ [45]; GPx mainly utilizes glutathione (GSH) as a reducing agent, and catalyzes the accelerated reaction of H_2_O_2_ to produce H_2_O [52]. MT contains a large number of cysteine residues, which are rich in sulfhydryl groups (-SH). The -SH readily reacts with and neutralize free radicals, such as hydroxyl radical (·OH) and $O_{2}^{\cdot -}$ [53]. In addition, MT could bind various ions (e.g. zinc, copper), which contribute to antioxidant defense by preventing these metals from participating in Fenton-like reactions that generate harmful free radicals [54]. (2) Non-enzymatic ROS-scavenging defenses include vitamins, *β*-catenin, coenzyme Q, selenium and zinc. Vitamin C reduces -SH enzymes and maintains their catalytic activity [55]; Coenzyme Q or zinc could scavenge free radicals by transferring electrons [56]. Enzymatic and non-enzymatic systems work together to maintain ROS homeostasis in the body. In addition, nuclear factor kappa-B (NF-*κ*B) is a redox-regulated transcription factor that controls cytokine production and cell survival. In fact, ROS overproduction impacts key pathways leading the inflammatory response such as the tumor necrosis factor receptor-alpha (TNF-*α*)-tumor necrosis factor receptor-1 (TNFR1) pathway, which balances cell survival, apoptosis and necroptosis through regulation of NF-*κ*B and, in turn, controls ROS signaling in immune cells [57].

### Bone formation

As previously stated, the metabolism and turnover rates of the jawbone and alveolar bone are the most active in the skeletal system. Notably, stem cell and OB activity in the jawbone and alveolar bone is distinct from that of other bone tissues. Jawbone-derived mesenchymal stem cells (m-BMSCs) have higher multiplication capacity, more pluripotency marker expression and higher osteogenic differentiation capacity than tibia-derived mesenchymal stem cells (t-BMSCs). Under pathological conditions such as oophorectomy and malnutrition, bone loss in the mandible was much lower than in the proximal tibia; Microarray analysis also showed that m-BMSCs had phenotypic characteristics more favorable to craniofacial bone regeneration than t-BMSCs [58]. Periodontal stem cells (PDLSCs) could differentiate into OBs and fibroblasts, support periodontal tissue maintenance and alveolar bone regeneration, and show better proliferative and osteogenic capacity than ilium-derived mesenchymal stem cells [59].

During oral bone repair, a high bone turnover rate promotes OB and OC activity, and promotes the production of more energy required for cellular metabolism, which leads to enhanced respiration or abnormal function of mitochondria, and electron leakage and more H_2_O_2_ generation during oxidative phosphorylation of the electron transport chain [44]. In addition, the metabolism of oral microbiome affects the local oxidative microenvironment, and macrophages produce massive ROS to prevent pathogenic bacteria during interaction with a host with high metabolic turnover [60]. ROS levels and redox balance have a direct impact on bone production efficiency and quality. Therefore, this section will explain the relationship between ROS and the main bone formation processes, including stem cell differentiation, OB activation and bone matrix mineralization.

#### Osteogenesis differentiation of mesenchymal stem cells

MSCs play a crucial role in bone formation, especially during the early stages of osteogenesis. Studies have shown that ROS levels have a bidirectional regulatory effect in this process. Appropriate levels of ROS can regulate the proliferation, differentiation and function of MSCs through multiple signaling pathways including Wnt/*β*-catenin, Notch and transforming growth factor-*β* (TGF-*β*). In this process, ROS activate a series of key transcription factors that promote the differentiation of MSCs into OBs, thereby promoting bone formation [61]. These signaling pathways not only regulate osteogenesis but also play a critical role in MSCs’ self-renewal and fate determination.

However, excessive ROS levels have a negative impact on MSCs function. High levels of ROS can block the phosphoinositide 3-kinase (PI3K)/protein kinase B (AKT) pathway and trigger autophagy, a process regulated by the PI3K family proteins. The PI3K family plays an important role in cell proliferation, differentiation, survival and glucose transport [62]. Excessive ROS can also damage cellular DNA, proteins and cell membranes, causing oxidative damage that reduces stem cell proliferation, impairs their differentiation potential, and may even lead to cell death [11, 32]. Moreover, prolonged high concentrations of ROS not only exacerbate oxidative stress in stem cells but may also lead to MSC exhaustion, limiting the repair and regeneration capacity of bone tissue. Research has shown that excessive ROS can activate aging-related genes, such as *p*53 and *p*16INK4a, which are closely related to MSCs aging. Aged MSCs lose their regenerative potential, impairing bone repair function and ultimately affecting the self-repair and regeneration capacity of bone tissue [63]. Therefore, the fine regulation of ROS levels is crucial for maintaining MSCs function and ensuring the normal development and repair of bone tissue.

#### Osteoblast activation and bone matrix mineralization

OBs are critical cell types in the process of bone development, primarily responsible for the synthesis and mineralization of bone matrix. Physiological ROS are typically maintained in the nanomolar (nM) range. Specifically, H_2_O_2_ is found in the 1–100 nM range under normal conditions [64]. In high-turnover oral bone tissue, the differentiation and function of OBs are particularly active. This process requires the upregulation of mitochondrial respiration in MSCs or OB precursors to enhance adenosine triphosphate (ATP) production, thereby providing more energy and increasing local endogenous ROS levels [65]. Evidence suggested that moderate to high ROS peaks could stimulate OB differentiation at the stage of distraction in distraction osteogenesis. However, declining ROS levels to baseline during the consolidation stage of bone remodeling was critical for supporting matrix deposition and mineralization [66]. When ROS levels exceed the capacity of the antioxidant system, triggering an oxidative stress response, extracellular signal-regulated kinase (ERK1/2), c-Jun N-terminal kinase (JNK) and p38 mitogen-activated protein kinase (MAPK) in the MAPK pathway are activated [65]. These signaling molecules can induce OB apoptosis [67]. Meanwhile, oxidative stress also inhibits OB differentiation and activity through the Wnt/*β*-catenin signaling pathway, thereby reducing the formation of mineralized matrix and the accumulation of new bone [68].

To cope with oxidative stress, the forkhead box O (FOXO) family of transcription factors (including FOXO1, FOXO3 and FOXO4) in OBs and osteocytes play an important role. These transcription factors stimulate bone formation by enhancing the expression of antioxidant enzymes [69]. However, oxidative stress can inhibit FOXO's transcriptional activity by phosphorylating AKT and ERK, increasing the interaction between FOXO and *β*-catenin, thus suppressing the Wnt/*β*-catenin signaling pathway and inhibiting osteogenesis [69, 70]. This indicates that, although the FOXO family has a role in defending against oxidative stress, its effectiveness in responding to oxidative stress still needs improvement.

Furthermore, excessive accumulation of ROS in OBs and inhibition of the Wnt/*β*-catenin signaling pathway result in impaired bone integration in hyperlipidemic mice under a high-fat environment. Using the ROS antagonist N-acetylcysteine (NAC) can reactivate the Wnt/*β*-catenin signaling pathway, significantly improving osteogenic ability [71]. This finding further emphasizes the potential therapeutic value of ROS regulation strategies in OBs.

### Bone resorption

Compared to long bones, mandibular bone marrow contains fewer OC precursors, mainly myeloid progenitor cells, and has a lower ratio of RANKL/OPG; mandibular OCs also express higher levels of anti-apoptotic genes and capture more anti-bone resorption drugs, making them more vulnerable [58]. However, mechanical stress increases RANKL-producing cells in periodontal tissues under pathological conditions of periodontitis and accelerates alveolar bone resorption [24]. Given the unique characteristics of oral bone tissue, this section will explore the physiological process of bone resorption from the perspective of OC differentiation and OC activation, and elucidate the role of ROS in regulating these processes.

#### Osteoclast differentiation and activation

**Figure 3.**
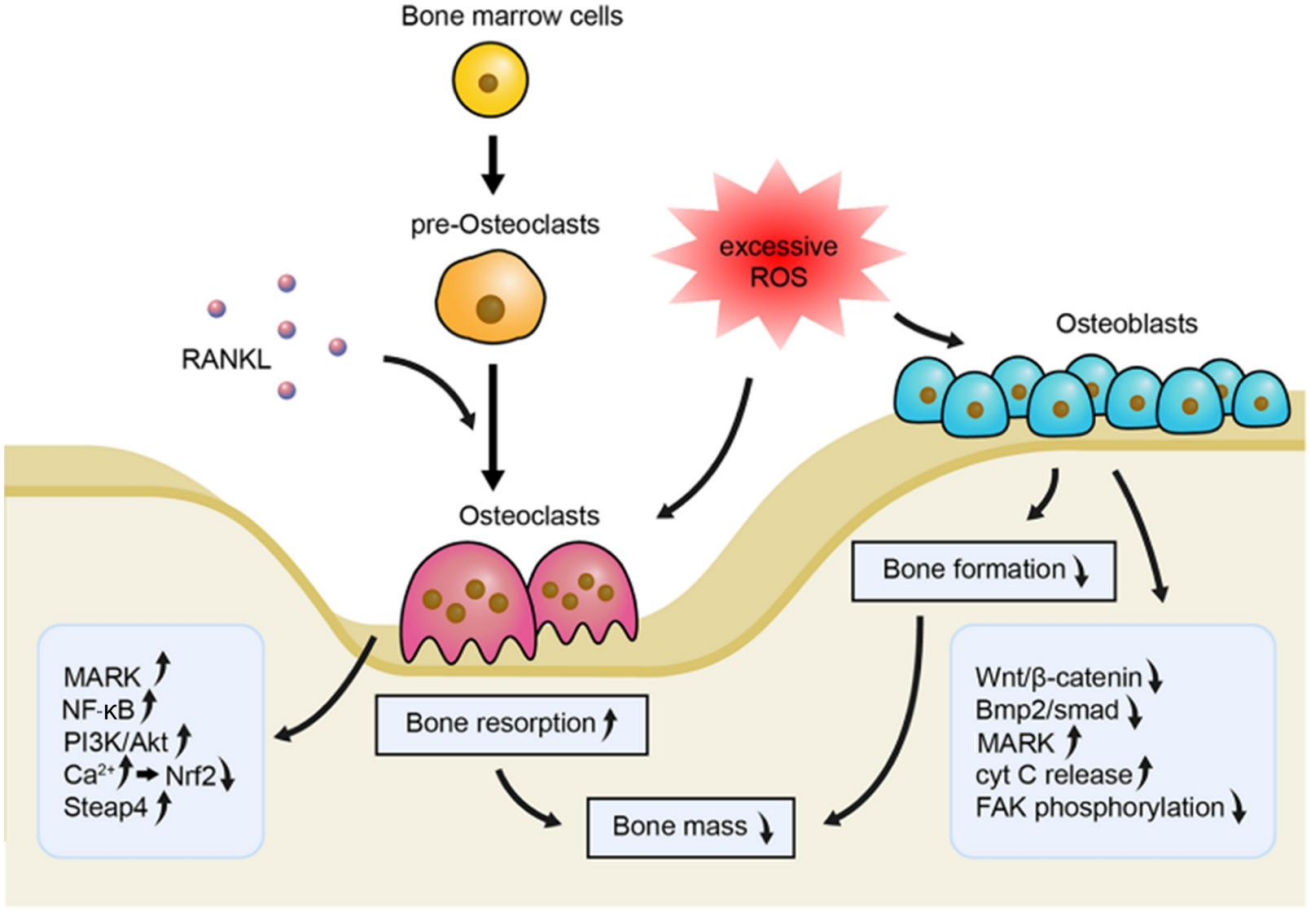
ROS signaling cascades regulate osteoclast and osteoblast. Excessive ROS promotes bone resorption through stimulating MARK, NF-*κ*B, PI3K/Akt, Steap4 signaling and inhibiting Nrf2 signaling, while it inhibits bone formation through suppressing Wnt/*β*-catenin, Bmp2/smad signaling, FAK phosphorylation and stimulating MARK and cyt C releasing. PI3K/Akt: Phosphoinositide 3-kinase/protein kinase B pathway. Nrf2: Nuclear factor E2-related factor 2. Steap4: Six transmembrane epithelial antigen of the prostate 4. Wnt: Wingless-related integration site. Bmp2/smad: Bone morphogenetic protein 2/Drosophila mothers against decapentaplegic protein pathway. cyt C: Cytochrome c. FAK: Focal adhesion kinase. Adapted with permission from Ref. [68]. Copyright 2020, Oxford University Press.

OCs are the prime bone resorptive cells, and local stimulation of their activity is an essential requirement for oral bone loss. The differentiation of OC precursors to mature OCs depends on the presence of RANKL, a member of the tumor necrosis factor family [72]. Under normal physiological settings, OBs block the transformation of OC precursors into OCs *via* regulating the OPG/RANKL/RANK signaling pathway. Under ROS targeting, RANKL activates the expression of MAPKs, PI3K and NF-*κ*B signaling molecules in the downstream pathway, which is a critical step in the induction of OC differentiation [73]. In a diabetic osteoporosis model, high glucose conditions induced ROS production and expression of MAPKs, NF-*κ*B and nucleotide-binding oligomerization domain-like receptor protein 3 (NLRP3) inflammasomes, which facilitated OC differentiation and bone resorption [74]. ROS regulates multiple signaling pathways to increase OC activity during normal bone metabolism. Moderate ROS levels activate MAPK, PI3K/Akt and other signaling pathways, maintaining moderate metabolic activity and bone resorption capacity of OCs [73] (Figure 3). In addition, the oxidative stress state is highly sensitive to RANKL/OPG expression, which promotes OB differentiation and activity by activating protein kinases like JNK and altering certain transcription factors. This results in an increase in the RANKL/OPG ratio by up-regulating RANKL expression and down-regulating OPG expression [65]. Furthermore, the ROS-mediated OC activation pathway also includes the triggering receptor expressed on myeloid cells-2 (TREM2) pathways [75]. Wen *et al.* reported that TREM2 expression was significantly upregulated in the alveolar bone of periodontitis patients, and OC-specific TREM2 conditional knockout periodontitis model mice exhibited reduced alveolar bone resorption. This study also found that the TREM2-dependent cascade was a significant signaling pathway of ROS-mediated osteoclastogenesis, suggesting that the TREM2-mediated ROS signal amplification cascade is essential in periodontitis osteoclastogenesis [76].

It is, thus, clear that moderate levels of ROS are critical for the redox equilibrium and bone-associated cells. Therefore, it is also necessary to develop ROS-regulating materials and address targeting problems for oral bone regeneration.

## Applications of reactive oxygen species-regulating biomaterials in oral bone regeneration

ROS-regulating biomaterials serve as a promising therapeutic approach due to their distinctive ability to mitigate oxidative stress within tissue microenvironments. These materials exhibit enzyme catalysis and/or chemical degradation properties, rendering them highly attractive for applications in tissue regeneration and disease therapy. However, some materials exhibit multiple functional mechanisms. To avoid reclassification, we categorized ROS-regulating biomaterials according to their primary functional mechanism: (1) Enzymatic biomaterials, which load with natural enzymes or mimic the activity of natural enzymes, facilitate the elimination of ROS through the catalysis process; (2) Direct ROS scavenging biomaterials reduce ROS through non-enzymatic mechanisms. While both ultimately reduce ROS levels, this classification highlights the mechanistic distinction between catalytic and noncatalytic scavenging pathways. This section will provide an overview of the mechanism underlying ROS-regulating biomaterials and recent advances in their application for oral bone regeneration treatment.

### Enzymatic biomaterials

According to the different active ingredients, enzymatic biomaterials are divided into two categories: (1) Biomaterials loaded with natural enzymes; (2) Biomaterials that mimic natural enzymes (Table 1).

**Table 1.** The application of the enzymatic biomaterials

Types	Materials	Antioxidant core materials	Observed effects	Key mechanism	Ref
Natural enzyme-based biomaterials	CAT and poly(D, L-lactide-co-glycolide) (PLGA) microspheres containing perfluorocarbons and poly(propylene sulphide)	CAT	Degrade hydrogen peroxide and enhancing bone defect repair	Natural enzymes like CAT show high sensitivity and clearance efficiency for ROS	[77]
ROS-scavenging mimetic enzyme-based biomaterials	Cobalt oxide (CoO)-loaded iridium (Ir) nanozymes	CoO	Exhibiting catalytic redox properties for ROS scavenging and promoting periodontitis therapy	These nanozymes exhibit the catalytic ability of natural ROS-scavenging enzymes and could be modified based on ROS scavenging requirements	[78]
	MIL-47(V)-F (MVF) nanozyme	Vanadium	Mimicking GPx to prevent cells from oxidative damage and promoting periodontitis therapy		[79]
	Platinum clusters equipped on carbon nitride frameworks	Platinum clusters equipped on carbon nitride frameworks	Mimicking CAT to prevent cells from oxidative damage and promoting periodontitis therapy		[80]
	Nano-hydroxyapatite with a supramolecular network of cerium ion-coordinated tannic acid (HA@Ce-TA)	Ce ions	Mimicking SOD/CAT to eliminate intracellularly excessive ROS and promoting infectious bone defect therapy		[81]
	Composites incorporating doping Ce nanoparticles into mesoporous bioactive glasses	Ce nanoparticles	Promoting mandibular bone regeneration		[82]
	Composites incorporating doping Ce nanoparticles into modifying 3D printed scaffolds	Ce nanoparticles	Repairing bone defect induced by oxidative stress		[83]
	CeO_2_ coated on the surface of mesoporous silica and modified with polyethylene glycol nanoplatforms	CeO_2_	Preventing H_2_O_2_-induced oxidative stress injury and promoting oxidative stress-induced periodontal disease therapy		[84]
	photosensitizer chlorin e6 (Ce6) coated on nano-CeO_2_	Nano-CeO_2_	Removing residual ROS and promoting periodontitis therapy		[85]
	Molybdenum (Mo)-containing bioactive glass-ceramic scaffolds	Mo ions	Inhibiting OC differentiation by decreasing the activity of OC mitochondrial biogenesis and the production of ROS, repairing alveolar bone		[86]
	Mo-based polyoxometalate nanoclusters encapsulated into gelatin methacrylate (GelMA)	Mo-based polyoxometalate nanoclusters	Scavenging excess ROS and enhancing bone regeneration by activating the PI3K/Akt signaling pathway		[87]
	chitosan/hydroxyapatite nanowires/polylactic acid barrier membranes loaded with manganese dioxide (MnO_2_) nanosheets	MnO_2_	exhibiting efficient catalytic properties for the decomposition of H_2_O_2_ and contributing to composite barrier membrane-mediated GBR		[88]
	Catechol-modified hydrogel incorporated poly(vinyl alcohol), 3,4-dihydroxy-D-phenylalanine (DOPA) and MnO_2_ nanoparticles	MnO_2_ nanoparticles	Exhibiting oxidation and reduction catalytic capabilities and promoting periodontitis therapy		[89]

#### Natural enzyme-based biomaterials

Natural enzymes with broad-spectrum capabilities have been shown to be effective biotherapeutic agents for numerous disorders caused by dysregulation of the ROS scavenging systems [90]. For instance, ROS are converted to H_2_O and O_2_ by a cascade process involving SOD and CAT [18]. Natural enzymes are highly specialized and substrate-specific, resulting in high sensitivity and clearance efficiency for ROS. Nevertheless, natural enzymes present several challenges, including storage difficulties, instability and a short *in vivo* half-life [78]. Furthermore, their clinical translation is hindered by limitations related to delivery mode, dose and treatment duration, which often restrict their therapeutic efficacy [91]. To optimize the utilization of natural enzymes, researchers have developed a range of enzyme-based biomaterials. For example, Sun *et al.* engineered a smart-responsive oxygen-releasing hydrogel that enzymatically cleaved ROS into oxygen by delivering CAT within the hypoxic microenvironment of bone defects. The resulting oxygen-rich microenvironment promoted neovascularization, inhibited OC activity and significantly enhanced bone regeneration. Specifically, CAT and poly(D, L-lactide-co-glycolide) (PLGA) microspheres containing perfluorocarbons and poly(propylene sulphide) were co-loaded into liposomes and finally incorporated to a gelatin methacrylate (GelMA) hydrogel. The hydrogel released CAT to degrade H_2_O_2_ to produce oxygen under low oxygen conditions, triggered by excess ROS, enabling a sustained release oxygen for over two weeks. The dual encapsulation enhanced CAT stability and provided controlled release [77].

#### Reactive oxygen species-scavenging mimetic enzyme-based biomaterials

**Figure 4.**
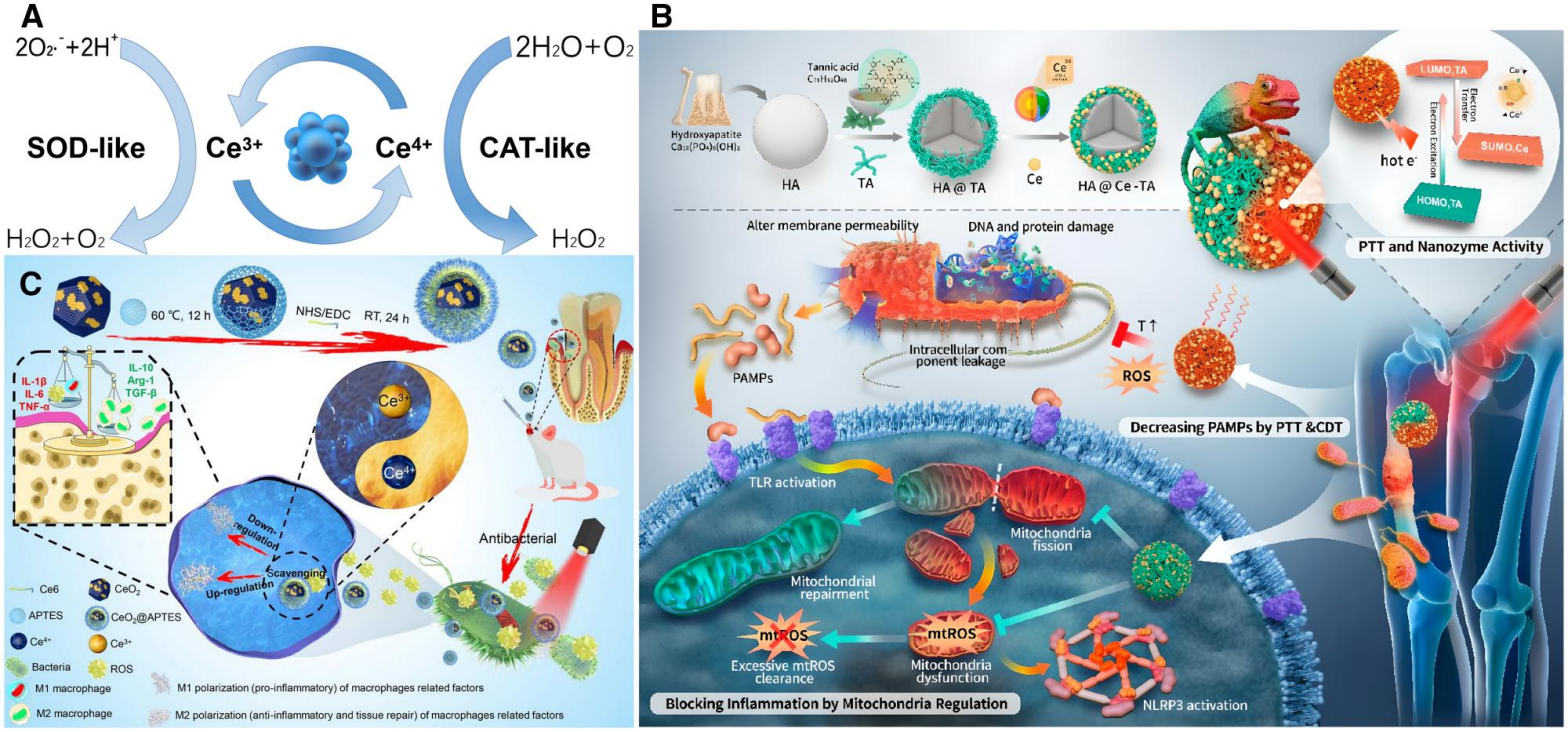
Representative ROS-scavenging mimetic enzyme-based biomaterials and their application. (**A**) Mechanism of ROS removal by CeO_2_ NPs. Ce^3+^ on the surface transforms superoxide radicals into oxygen and H_2_O_2_, while Ce^4+^ scavenges H_2_O_2_ and generates oxygen and water, ultimately eliminating ROS. Reproduced with permission from Ref. [40]. Copyright 2023, BioMed Central. (**B**) Schematic illustration of mitochondrial homeostasis regulation for macrophage by the HA@Ce-TA NPs. HA@Ce-TA acted as peroxidase mimics to generate ROS, acted as oxidase mimics to scavenge ROS and blocked inflammation by mitochondria regulation, therefore, performing anti-infective, antioxidant and anti-inflammatory effects. Reproduced with permission from Ref. [81]. Copyright 2023, American Chemical Society. (**C**) Schematic illustration of CeO_2_@Ce6 nanocomposite in synthesis, the antibacterial mechanism and modulating the polarization of macrophages for the treatment of periodontal diseases. Reproduced with permission from Ref. [85]. Copyright 2020, Elsevier Ltd.

As previously stated, natural enzymes’ poor stability and short half-life severely limit their applications. Therefore, mimetic enzymes have been developed. These mimetic enzymes are designed to mimic the natural ROS-scavenging enzymes *in vivo* (Figure 4) [92]. Among these, artificial nanozymes have emerged as promising alternatives due to their enhanced stability, versatility and tunable activity, enabling them to effectively eliminate to complement, restore or correct enzyme activity to eliminate residual ROS in disease conditions [93, 94]. Nanozymes exhibit high sensitivity to ROS and can be engineered to enhance their sensitivity by modifying their size, shape and composition, making them highly effective at low concentration of ROS [95]. The modified nanozymes could exhibit catalyzing at micromolar (μM) levels of H_2_O_2_ concentration and reached a maximum reaction rate of 300 μM s^−1^ [96].

#### Cobalt oxides

Xie *et al.* developed cobalt oxide (CoO)-loaded iridium (Ir) nanozymes, in which CoO acted as a stable carrier with high redox potential (Co^3+^/Co^2+^). Furthermore, the chemical coupling between Ir nanoclusters and CoO substrates and strong interfacial charge transfer from cobalt (Co) to Ir sites may promote the stabilization and catalytic redox properties of this CoO-Ir-based artificial antioxidases [97]. Their study demonstrated that Co to the Ir worked synergistically, forming a stable chemical coupling that facilitated charge transfer from the Co site to the Ir site, thereby promoting its original catalytic redox properties for ROS scavenging, effectively guarding OBs [78]. In addition, researchers developed nanozymes that mimic different natural enzymes, all of which could effectively protect cells from oxidative damage, such as the vanadium node MIL-47(V)-F (MVF) nanozyme that mimics GPx and platinum clusters nanozyme that mimics the Fe-N ligand structure of CAT [79, 80]. This targeted approach not only reduced inflammation but also promoted periodontal regeneration by activating the nuclear factor E2-related factor 2 (Nrf2)/heme oxygenase-1 (HO-1) and PI3K/Akt pathways, which were crucial for osteogenic differentiation [79].

#### Cerium oxides

Cerium (Ce) is one of the most abundant rare earth elements, exhibiting redox properties due to its electronic configuration [Xe]4f^1^5d^1^6s^2^ and the ability to form stable +3 or +4 oxidation states [98]. Chen *et al.* wrapped nano-hydroxyapatite with a supramolecular network of cerium ion-coordinated tannic acid (HA@Ce-TA). Ce ions mimicked peroxidase/oxidase to generate ROS under extracellular acidic conditions, and mimicked SOD/CAT to eliminate intracellularly excessive ROS. In addition, tannic acid (TA) acts as antioxidant due to its rich polyphenol groups (Specific mechanisms are described in section Biomaterials based on noncleavage chemistry), which targets mitochondria without utilizing mitochondrial membrane charge. This inflammatory cascade response targeted to regulating mitochondrial homeostasis accelerated repair infected bone defects [81]. Composites incorporating doping Ce nanoparticles into mesoporous bioactive glasses or modifying 3D printed scaffolds have shown promise in repairing bone defects induced by oxidative stress [82, 83]. Nanoscale Ce oxides possess a higher surface area ratio, leading to increased oxygen vacancies, which enhances their reactivity with CeO_2_ redox equilibrium [99]. CeO_2_ coated on the surface of mesoporous silica and modified with polyethylene glycol nanoplatforms produced significant therapeutic benefits in H_2_O_2_-induced oxidative stress injury and oxidative stress-induced periodontal disease rat models [84]. The photosensitizer chlorin e6 (Ce6) was coated on nano-CeO_2_ to exert both anti-bacteria and anti-inflammation effects through a bidirectional regulatory effect. The first stage was antimicrobial photodynamic therapy (aPDT), in which red light irradiation generates ROS to kill pathogenic bacteria, and then, the residual ROS was removed by nano-CeO_2_ to eliminate the inflammation [85].

#### Molybdenum oxides

Beyond the previously discussed redox nanomaterials and designs, molybdenum (Mo) functions as a catalytic center for a variety of enzymes and plays an important role in maintaining metabolic homeostasis by catalyzing redox reactions [86]. The ROS scavenging ability of Mo-based antioxidant nanozymes is mainly due to the transfer of electrons from the lower valence to the upper valence (Mo^4+^ to Mo^6+^) [100]. Mo-containing bioactive glass-ceramic scaffolds have been shown to inhibit OC differentiation by decreasing the activity of OC mitochondrial biogenesis and the production of ROS [86]. Liao *et al.* developed an injectable hybrid hydrogel with antioxidative properties by encapsulating Mo-based polyoxometalate nanoclusters into GelMA. The Mo-based polyoxometalate nanoclusters in this hydrogel were released at the bone defect in diabetic models, persistently scavenging excess ROS and enhancing bone regeneration by activating the PI3K/Akt signaling pathway [87].

#### Manganese dioxide

By scavenging H_2_O_2_, generating O_2_, alleviating inflammation and modulating immune responses, manganese dioxide (MnO_2_) contributes to composite barrier membrane-mediated guided bone regeneration (GBR). Additionally, MnO_2_ exhibits efficient catalytic properties for the decomposition of H_2_O_2_ [88]. Hu *et al.* prepared a catechol-modified hydrogel incorporating poly(vinyl alcohol) (PVA), 3,4-dihydroxy-D-phenylalanine (DOPA) and MnO_2_ nanoparticles. The catechol moiety of DOPA could be oxidized to a quinone structure, which subsequently interacted with the sulfhydryl and amine groups in proteins to achieve solid adhesion. The simultaneous presence of multivalent Mn ions endowed MnO_2_ nanoparticles with oxidation and reduction catalytic capabilities. This combination enhances hydrogels’ efficacy in treating periodontitis [89].

### Direct reactive oxygen species scavenging biomaterials

#### Biomaterials based on noncleavage chemistry

**Figure 5.**
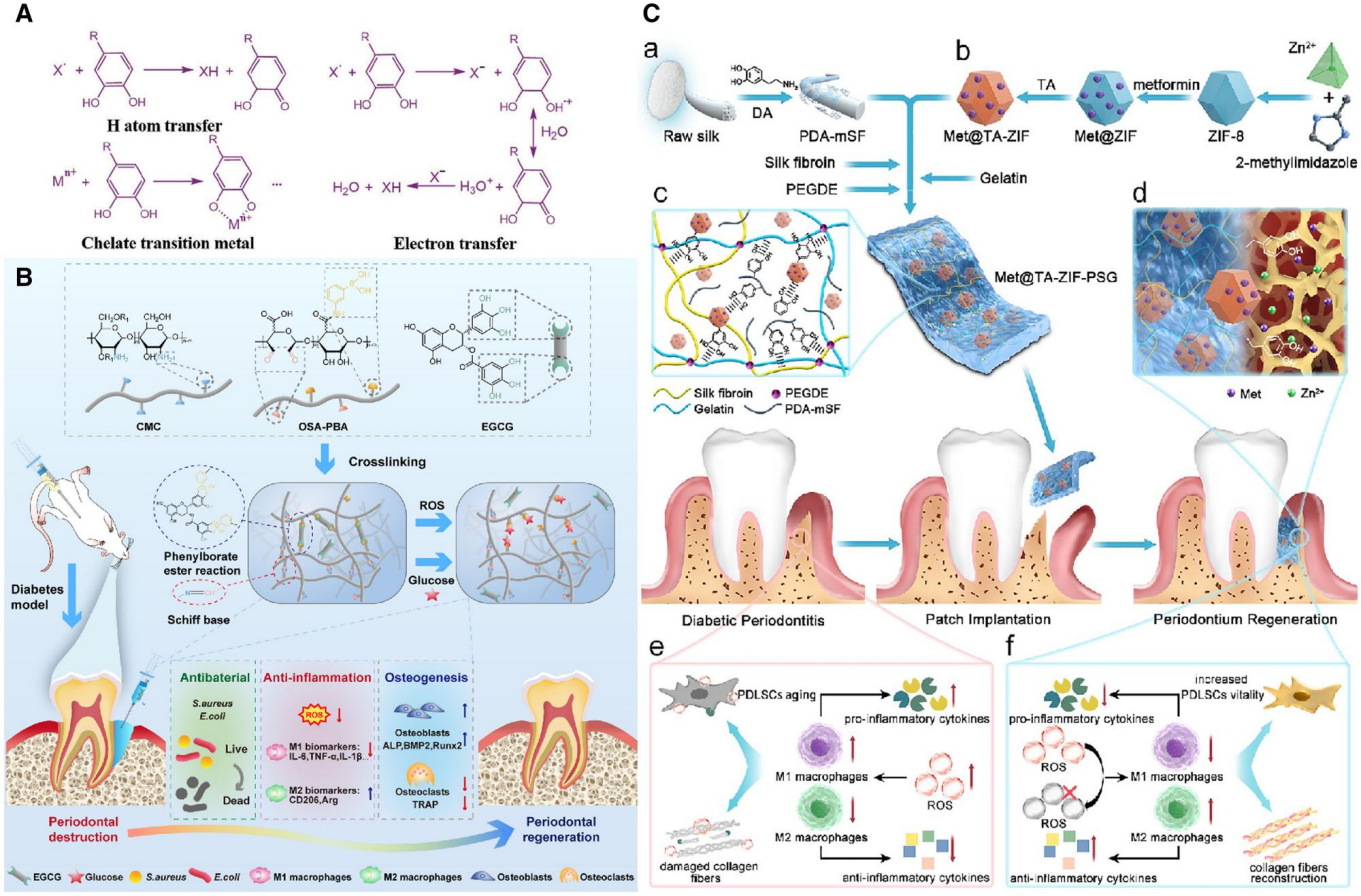
Representative polyphenol-based biomaterials and their application. (**A**) The free ROS scavenging mechanism of polyphenol-based ROS materials. Polyphenols possess benzene ring bound hydroxyl groups that could donate one hydrogen atom or a single electron to the ROS, stabilizing the reactive species. Adapted with permission from Ref. [106]. Copyright 2020, John Wiley and Sons Ltd (**B**) Schematic illustration of the OSA–PBA/CMC + EGCG hydrogel with ROS/glucose-triggered drug release for chronic periodontitis in diabetic rats. This hydrogel has ROS/glucose-triggered release of EGCG, showing favorable antioxidant and anti-inflammatory properties and providing an ideal immune microenvironment. Reproduced with permission from Ref. [107]. Copyright 2024, Royal Society of Chemistry. (**C**) Schematic illustration of preparation and applications of the Met@TA-ZIF-PSG patch. The PDA-mSF endows the patch with ROS-scavenging ability and anti-inflammatory activity, reducing the inflammatory response by suppressing M1 macrophage polarization. Reproduced with permission from Ref. [108]. Copyright 2023, American Chemical Society.

Polyphenols are characterized by the presence of multiple phenolic rings. These compounds, acting as exogenous ROS scavenger agents, are extensively utilized and have been employed in the treatment and prevention of various disorders associated with ROS. Numerous studies indicated that polyphenols could directly neutralize ROS [101]. Existing research indicated that polyphenols could undergo sacrificial reactions with a range of free radicals, including ·OH, $O_{2}^{\cdot -}$, alkoxy and peroxy radicals as well as non-free radicals such as peroxynitrite (ONOO^-^) and hypochlorite (ClO^-^) [102]. The mechanism underlying the ROS scavenging activity of polyphenols can be categorized into two distinct pathways. The first mechanism involves the hydroxyl groups attached to the benzene ring, which play a primary role in polyphenol-mediated ROS scavenging. These hydroxyl groups can stabilize reactive species by donating either single electron or a hydrogen atom to the ROS [103]. One key process is hydrogen atom transfer, in which polyphenols (ROH) neutralize free radicals (X^·^) by donating hydrogen atoms [102]. The second mechanism is single-electron transfer from ROH to X^·^ with formation of a stable radical cation ROH^+^ [104]. Consequently, polyphenols produce phenolic groups that interact with another radical to form a stable quinone structure. Depending on the pattern and concentration of hydroxyl substituents, certain polyphenols—such as quercetin and epigallocatechin gallate (EGCG) can also function as pro-oxidants, which may be explained by this mechanism [103]. Furthermore, in response to oxidative stress, the hydroxyl groups attached to the benzene ring can also bind transition metals. A study demonstrated that polyphenols such as quercetin, resveratrol and plant extract imposed antioxidant effect within 2–20 min [105]. In conclusion, the protective effects of polyphenols in biological systems are largely attributed to their ability to bind metal catalysts and transfer electrons from free radicals (Figure 5; Table 2).

**Table 2.** The application of direct ROS scavenging biomaterials

Types	Materials	Antioxidant core materials	Observed effects	Key mechanism	Ref
Biomaterials based on noncleavage chemistry	Ellagic acid (EA)	EA	Reducing gingival oxidative stress and decreasing inflammatory indicators, as well as reducing alveolar bone resorption	These hydroxyl groups (ROH) can stabilize reactive species (X^·^) by donating either single electron or a hydrogen atom to the ROS	[109]
	Epigallocatechin-3-gallate (EGCG)	EGCG	Enhancing the expression of SOD, mitigating oxidative stress and inflammation by modulating the Nrf2/HO-1/NLRP3/NF-*κ*B p65 signaling pathway within periodontitis model		[110]
	Silk fibroin/polycaprolactone core-shell nanofibers loaded with EGCG	EGCG	Exhibiting potent antioxidant properties and promoting adhesion, proliferation and osteogenic differentiation of MC3T3-E1 cells		[111]
	Oxidized sodium alginate and carboxymethyl chitosan hydrogel loaded with EGCG	EGCG	Exerting the functions of antioxidant and anti-inflammatory and promoting diabetic periodontitis therapy		[107]
	The layer-by-layer combined with polyphenols, 4-(bromomethyl)phenylboronic acid and minocycline	EGCG	Exhibiting free radical scavenging capabilities and promoting periodontal regeneration		[112]
	Metal–phenolic networks and bone morphogenetic protein 2 (BMP2) coated with gold nanoparticles	TA	Exhibiting antioxidant properties, blocking the initiating factors of periodontitis and attenuating the excessive immune response		[113]
	Thermosensitive beta-glycerophosphate chitosan/collagen hydrogels incorporated quercetin	Quercetin	Mitigating cellular damage caused by free radical molecules and promoting growth viability and osteogenic differentiation of hPDLSCs		[114]
	Injectable quercetin-loaded bioglass microsphere hydrogel	Quercetin	Reducing oxidative stress by modulating m6A alterations in Per1 and promoting periodontal regeneration		[115]
	Quercetin	Quercetin	Reduces impaired osteogenesis in hPDLSCs by inhibiting the NF-*κ*B/NLRP3 inflammasome pathway under TNF-*α*-induced inflammatory conditions		[116]
	Hyperoside	Glycoside derivatives of quercetin	Promote osteogenic differentiation under inflammation by simultaneously inhibiting NF-*κ*B activation, *β*-catenin protein expression and ROS production		[117]
	Chlorogenic acid (CGA)	CGA	Inhibiting oxidative stress by promoting the nuclear translocation of Nrf2 and decreasing the loss of alveolar bone in periodontitis		[118]
	CGA	CGA	Attenuating inflammation and suppressing oxidative stress in LPS-induced HGFs possibly through CysLT1R/Nrf2/NLRP3 signaling		[119]
	5 μM curcumin	Curcumin	Protecting hPDLSCs against oxidative stress injury *in vitro* and improving the transplanted hPDLSCs cell viability and bone repair *in vivo*		[120]
	GelMA microspheres encapsulated with iron-curcumin nanoparticles and modified with a polydopamine (PDA) layer and minocycline hydrochloride	Curcumin	Promoting ROS scavenging by modulating the Nrf2 signaling pathway while inhibiting the activation of the NLRP3 inflammasome and promoting periodontitis therapy		[121]
	Tocopherol	Tocopherol	Protecting BMSCs from oxidative stress damage *via* the inhibition of ferroptosis through the PI3K/Akt/mTOR pathway		[122]
	Resveratrol	Resveratrol	preventing ligature/LPS-mediated alveolar bone loss in rats, attenuating the production of inflammation-related proteins and suppressing LPS-mediated decreases in HO-1 and Nrf2 levels in the inflamed periodontal tissues		[123]
	N-acetyl cysteine (NAC)-loaded nanotube titanium implants	NAC	Decreasing inflammatory responses and reduced oxidative stress-related defense, enhancing osseointegration		[124]
	NAC	NAC	Enhancing osteogenesis *via* PI3K/Akt/ROS signaling		[125]
	Polymer nanoparticles loaded with NAC	NAC	regulating the osteoimmune microenvironment and protecting stem cells from oxidative stress injury for bone regeneration through MAPK/NF-*κ*B phosphorylation pathway		[126, 127]
	Polydopamine (PDA)-mediated ultralong silk microfiber and metformin-loaded zeolitic imidazolate framework incorporated into a silk fibroin/gelatin patch	PDA	Exhibiting ROS scavenging capacity and anti-inflammatory activity, improving periodontal ligament reconstruction in diabetic periodontitis		[108]
	PDA-mediated graphene oxide (PGO) and hydroxyapatite nanoparticle-incorporated conductive alginate/gelatin scaffold	PGO	Showing good conductive, ROS scavenging, anti-inflammatory and immunomodulatory abilities on diabetic periodontal regeneration		[128]
	Degradable polyurethane membrane with Janus surface morphology integrated DA and Gemini quaternary ammonium salt	DA	Promoting cell adhesion and MSC growth as well as supporting mineralization and antioxidant properties, promoting periodontal regeneration		[129]
	Cell membrane coated PDA nanoparticles	PDA	Rescuing impaired hPDLSCs through antioxidant, anti-ferroptosis, anti-inflammatory and pro-osteogenic effects, promoting periodontitis therapy		[130]
	Biomimetic periosteum with poly(3-hydroxybutyric acid-*co*-3-hydrovaleric acid) polymer matrix, PDA-modified hydroxyapatite and barium titanate	PDA	ROS scavenging and promoting the polarization of macrophages to the M2 phenotype on osteogenesis		[131]
	BMP-2 loaded PDA/heparin nanoparticles	PDA	Depleting elevated ROS levels to protect cell viability against ROS damage, offering effective approach to promote bone repair		[132]
	Chitosan-catechol chelated the Ca^2+^ of nanohydroxyapatite and bonding type I collagen	Catechol	Maintaining redox balance between free radical generation and elimination, promoting periodontal regeneration		[133]
	sericin-hydroxyapatite nanoparticles and proanthocyanidins encapsulated in sericin/sodium alginate	Proanthocyanidins	Scavenging ROS and promoting the polarization of macrophages to the M2 phenotype, promoting periodontitis therapy		[134]
Biomaterials based on electrical conductivity	Aniline tetramer and glycine ethyl ester co-substituted polyphosphazene	Aniline tetramer	Exhibiting ROS scavenging effect, up-regulating cellular activities and speeding up new bone formation in rat calvarial defects	Organic conductive materials typically contain groups with *π*-electron structure (e.g., aromatic rings) or structural units capable of donating free electrons (e.g., pyrrole, aniline, etc.) Inorganic conductive materials typically driven by the free electrons in metals, the electronic band structure of semiconductors, or the conductive properties of carbon materials (e.g., *p* electrons in graphene)	[135]
	PLGA/Polyphosphazenes core–shell nanofibers	Polyphosphazenes	scavenging ROS, reducing inflammation, promoting differentiation and enhancing osteogenesis		[136]
	Amorphous silicon oxynitride (SiONx)	N/O ratio	enhancing the expression of cell-mediated bone markers in oxidative stress situations, as well as Nrf2 activity		[137]
	Injectable thermosensitive hydrogel incorporated black phosphorus (BP) nanoflakes and dl-3-*n*-butylphthalide (NBP)	BP	Protecting cells against ROS-induced damage and promoting macrophage M2 polarization, promoting periodontitis therapy		[138, 139]
	Injectable hydrogel composed of gelatin, Ti_3_C_2_T_*x*_ MXene nanosheets and poly-*L*-lysine	MXene nanosheets	Scavenging ROS, attenuating inflammatory responses and enhancing bone tissue regeneration for the treatment of periodontitis		[140]
Biomaterials based on ROS-induced bond cleavage	*β*-cyclodextrin—Tempol and phenylboronic acid pinacol ester conjugates	Phenylboronic acid	Periodontitis therapy	For the ROS-induced structural cleavage, ROS can react with various chemical groups such as phenylboronic acid, ester bonds and thioketals, hydrazone and disulfide linkages, leading to the structure cleavage and skeleton collapse	[141]
	Hyaluronic acid-phenylboronic acid pinacol ester conjugates	Phenylboronic acid	Periodontitis therapy		[142]
	Phenylboronic-acid-crosslinked poly(vinyl alcohol) and gelatin colloids hydrogel	Phenylboronic acid	Diabetic bone defects therapy		[143]
	Hydrogel integrated Carboxymethyl Chitosan, Dextran and 4-formylphenylboronic acid	4-formylphenylboronic acid	Releasing drug in response to ROS levels and providing favorable conditions for the improvement of bone regeneration microenvironment in periodontitis		[144]
	poly(thioketal) urethane (PTKUR) biomaterial scaffolds	Thioketone (TK)	Reducing ROS		[145]
	PTKUR	TK	Reducing ROS associated with bone defect		[146]
	Hydrogel contained thioketal linkers and ultraviolet (UV)-responsive norbornene groups conjugated with 8-arm poly(ethylene glycol) macromers	TK	Reducing ROS levels, promoting BMSC osteogenic differentiation and leading to effective repair of bone defects in diabetic model		[147]

#### Ellagic acid

Ellagic acid (EA) is a polyphenol compound renowned for its anti-inflammatory and antioxidant properties, found in a variety of fruits and medicinal plants. To evaluate the therapeutic impact of oral EA on periodontitis, rats with experimental periodontitis were administered EA through tube feeding. The results demonstrated a considerable improvement in gingival oxidative stress and inflammatory indicators, as well as a reduction in alveolar bone resorption during the recovery phase associated with experimental periodontitis [109]. These findings suggest that EA has therapeutic potential for the management of periodontitis.

#### Epigallocatechin gallate

EGCG is the most abundant and biologically active catechin polyphenol found in green tea. Qin *et al.* investigated the therapeutic effects of EGCG by administering it at a concentration of 200 mg/kg to rats with periodontitis. Their study revealed that EGCG significantly enhanced the expression of SOD in the periodontitis rats and effectively mitigated oxidative stress and inflammation within the rat periodontitis model by modulating the Nrf2/HO-1/NLRP3/NF-*κ*B p65 signaling pathway [110]. Additionally, Chen *et al.* developed EGCG-laden filipin protein/polycaprolactone (PCL) core-shell nanofibers using emulsion electrospinning. The EGCG-loaded membranes exhibited potent antioxidant properties and promoted the adhesion, proliferation and osteogenic differentiation of MC3T3-E1 cells [111]. In addition to bone defects caused by local factors in the oral cavity, EGCG is also being utilized to treat oral and maxillofacial bone defects associated with systemic diseases. The microenvironment of chronic periodontitis in diabetes mellitus was established by elevated glucose levels, multiple pro-inflammatory factors and excessive ROS production [148]. Feng *et al.* synthesized an injectable hydrogel with phenylboronic acid-functionalized oxidized sodium alginate (OSA-PBA), carboxymethyl chitosan (CMC) and EGCG. This hydrogel would release EGCG when triggered by ROS/glucose to exert the functions of antioxidant and anti-inflammatory [107]. Furthermore, Chen *et al.* employed a layer-by-layer assembly technique to combine EGCG, 4-(bromomethyl)phenylboronic acid, and minocycline (Mino) to prepare a novel bone repair barrier membrane with possessing antimicrobial activity and antioxidant properties. This membrane exhibited a ROS response mechanism for controlled EGCG release, and successfully promoted periodontal bone regeneration [112].

#### Tannic acid

TA is known for use in antioxidants, and there has been a renewed interest in novel engineered functional composites with TA modification. The modification process involves various interactions/reactions based on its diverse chemical structure, such as TA-based metal phenolic networks (MPN), contributed by abundant aromatic rings and hydroxyl groups [149]. MPN consisting of strontium and TA encapsulated around gold nanoparticles and loaded with bone morphogenetic protein 2 (BMP2), were constructed into multifunctional composites. The polyphenol-rich MPN structure exhibited significant antimicrobial and antioxidant properties, effectively blocking the initiating factors of periodontitis and attenuating the excessive immune response; in combination with BMP2, the MPN composite promoted osteogenesis and inhibited periodontitis-related bone loss [113].

#### Quercetin and its glycoside derivatives

Quercetin exhibits potential antioxidant activity due to the phenolic hydroxyl group and a double bond [150]. Premjit *et al.* designed a heat-sensitive *β*-glycerophosphate-chitosan/collagen hydrogel incorporating quercetin. This hydrogel achieved a sol-to-gel transition at 37°C, providing the requisite pore structure for cellular growth. Quercetin has been shown to promote growth viability and osteogenic differentiation of human periodontal ligament stem cells (hPDLSCs), while also mitigating cellular damage caused by free radical molecules [114]. Moreover, quercetin exerts its effects by modulating m6A alterations in Per1, which leads to a reduction in oxidative stress in orofacial MSCs *in vitro*, thereby influencing their osteogenic development [115]. Quercetin reduces impaired osteogenesis in hPDLSCs by inhibiting the NF-*κ*B/NLRP3 inflammasome pathway under TNF-*α*-induced inflammatory conditions [116]. Glycoside derivatives of quercetin, such as hypericin or 2-furyl-LIGRLO-amide trifluoroacetate, as FOXO1 agonists exert a protective effect on osteogenesis in PDLSCs and promote osteogenic differentiation under inflammation by simultaneously inhibiting NF-*κ*B activation, *β*-catenin protein expression and ROS production [117].

#### Chlorogenic acid

Chlorogenic acid (CGA) has a comprehensive antioxidant mechanism, is summarized as follows: (1) The polyhydroxy structure directly scavenges free radicals like superoxide anions and hydroxyl radicals; (2) Activate antioxidant signaling pathway like Nrf2 and regulate the expression level of related genes; (3) Directly regulates the activity of endogenous oxidase system and associated proteins like SOD and CAT [151]. CGA inhibits oxidative stress by promoting the nuclear translocation of Nrf2, increases the mitochondrial membrane potential, thereby decreasing the protease activity of porphyromonas gingivalis, decreasing the loss of alveolar bone in periodontitis and preventing inflammation [118]. Furthermore, the study by Huang *et al.* suggested that CGA could attenuate inflammation and suppress oxidative stress in lipopolysaccharide (LPS)-induced human gingival fibroblasts (HGFs), which was possibly through cysteinyl leukotriene receptor 1 (CysLT1R)/Nrf2/NLRP3 signaling [119].

#### Curcumin

Curcumin is a hydrophobic polyphenol and the main active ingredient of turmeric. The curcuminoid structure contains several functional groups, including the phenolic hydroxyl and methoxy groups [152]. These functional groups confer upon curcumin the potential ability of free radical scavenging and oxidative stress inhibiting. Tan *et al.* found that 5 μM curcumin pretreatment protected hPDLSCs against oxidative stress injury *in vitro* and improved the transplanted cell viability and bone repair *in vivo*. Furthermore, the results showed that curcumin activated the ERK pathway and upregulated runt-related transcription factor 2 (Runx2) expression, thereby protecting the osteogenic differentiation of hPDLSCs from oxidative stress damage [120]. However, the bioavailability and low solubility of the curcumin limit its therapeutic potential. Therefore, Xiu *et al.* synthesized fully water-soluble iron-curcumin coordination nanoparticles for periodontitis treatment by combining iron ions with curcumin, enhancing the bioavailability of the curcumin. By encapsulating the curcumin-included nanoparticle within GelMA microsphere, this material possessed outstanding ROS scavenging abilities by modulating the Nrf2 signaling pathway while inhibiting the activation of the NLRP3 inflammasome, offering a promising therapeutic strategy for treating periodontitis [121].

#### Tocopherol

Tocopherol is a hydrolysis product of vitamin E that has the potential to scavenge free radicals. The ROS scavenging mechanism involves the donation of the hydrogen atom from the hydroxyl group of the chromanol ring of tocopherol to ROS [153]. In clinical studies, tocopherol supplementation showed a modest benefit in managing periodontal disease, particularly in reducing clinical attachment levels and pocket depth [154]. Lan *et al.* found that tocopherol could protect bone marrow derived mesenchymal stem cells (BMSCs) from oxidative stress damage *via* the inhibition of ferroptosis through the PI3K/Akt/mammalian target of rapamycin (mTOR) pathway [122].

#### Resveratrol

Resveratrol is a naturally occurring polyphenol and stilbene derivative. The Sirt1 activator resveratrol inhibits human MSC senescence to encourage the creation of alveolar bone by promoting Bmi1 deacetylation and nuclear translocation [155]. In *vivo* studies, resveratrol prevented ligature/LPS-mediated alveolar bone loss in rats, attenuated the production of inflammation-related proteins and suppressed LPS-mediated decreases in HO-1 and Nrf2 levels in the inflamed periodontal tissues [123].

#### N-acetylcysteine

NAC exerts ROS-regulating effects mainly associated with the -SH, and the specific principles are as follows: (1) Hydrogen-donating effect of -SH: -SH in NAC is a highly reactive group that can neutralize free radicals by donating hydrogen atoms. For example, -SH are able to react with H_2_O_2_ and certain free radicals (e.g. ·OH, $O_{2}^{\cdot -}$) to produce stable products. (2) Reducing effect: NAC exhibits reducing properties that may promote the reduced state of antioxidant enzyme systems (e.g. GSH peroxidase, CAT, etc), thereby enhancing cellular resistance to oxidative stress. Notably, under conditions of reduced GSH levels, NAC can assist in restoring and maintaining the normal level of GSH, indirectly enhancing the antioxidant capacity. (3) Peroxyl radicals scavenging: NAC can also directly capture peroxyl radicals (ROO·) and other oxidizing radicals, forming stable compounds in the process. Through the activity of -SH, NAC can effectively scavenge these peroxides and harmful free radicals to reduce oxidative damage. NAC can be used to perform implant surface treatment [124] or dental follicular stem cell conditioned culture [125] or to construct a ROS-responsive nanoplatform [126, 127]. All of the treated implants demonstrated potent ROS-scavenging and bone regeneration-promoting properties.

When polyphenol monomers are utilized directly as ROS scavengers, the body’s ability to scavenge ROS is hampered by their instability and short half-life, which results in high dose toxicity and a number of negative effects. To improve the use of polyphenols, numerous researchers have tried to synthesize polymeric polyphenols or polyphenol-polymer affixes. The research on catechol and dopamine (DA) is representative of them.

#### Dopamine and polydopamine

In addition to its strong adhesive properties, DA also plays a significant role in reducing inflammation by scavenging ROS and mitigating the oxidative stress. The catechol moiety of DA is one of the main structural features responsible for its antioxidant action [156]. Polydopamine (PDA) is frequently utilized for surface modification of various substrates to enhance their reactivity [157]. Gong *et al.* designed silk fibroin/gelatin (SG) patches doped with PDA-mediated ultralong silk microfiber (PDA-mSF) and metformin (Met)-loaded zeolitic imidazolium framework (ZIF). The PDA-mSF endowed patches with ROS scavenging capacity and anti-inflammatory activity, effectively reducing inflammatory responses by suppressing M1 macrophage polarization. Moreover, the affinity of PDA facilitated improving periodontal ligament reconstruction [108]. In a similar vein, Li *et al.* designed conductive alginate/gelatin scaffolds doped with PDA-mediated graphene oxide (PGO) and hydroxyapatite nanoparticles, which promoted periodontal bone regeneration through modulating the diabetic inflammatory microenvironment. The PDA-induced PGO showed better electrical conductivity, ROS scavenging and anti-inflammatory ability, allowing the scaffolds to transmit endogenous signals to cells for bone regeneration [128]. He *et al.* incorporated bioactive DA into degradable polyurethane films, while Pan *et al.* developed nanoparticles loaded with PDA and Mino, both of which underscore the multifunctional potential of DA in chemical modification [129, 130]. Additionally, Liu *et al.* prepared a biomimetic periosteum using poly(3-hydroxybutyric acid-co-3-hydroxyvaleric acid) polymer matrix, antioxidant PDA-modified hydroxyapatite and barium titanate. This piezoelectric material generated an internal electric field under mechanical stress, which could separate electrons and holes. Then the electrons and holes undergo redox reactions with surrounding molecules such as water and oxygen to produce ROS. The incorporation of PDA not only improved the properties of cell adhesion and ROS-scavenging, but also acted as an immune regulator, promoting the polarization of macrophages to the M2 phenotype on osteogenesis [131]. Furthermore, Wu *et al.* demonstrated that PDA/heparin nanoparticles possessed ROS scavenging capabilities and depleted elevated ROS levels to protect cells viability, offering effective approach to promote bone repair [132].

#### Catechol and catechol couplers

As a common polyphenol subunit, catechol has been extensively incorporated into polymers as main, side and terminal chains through a variety of synthetic processes, including metal-chelate polymerization, oxidative polymerization, enzymatic polymerization and free radical polymerization, to achieve highly effective ROS scavenging capabilities. Chitosan-catechol couplings inspired by mussel adhesion proteins, exhibit excellent cell adhesion properties and ROS scavenging activity [133]. Building on this, Wu *et al.* then prepared cationic biopolymer scaffolds by chelating nanohydroxyapatite and combining it with collagen type I using CS-H as a cross-linking agent. The catechol’s inherent antioxidant abilities endowed this scaffold with the capability to maintain redox balance between free radical generation and elimination [133]. Similarly, Ming *et al.* synthesized sericin-hydroxyapatite nanoparticles through biomimetic mineralization and developed multifunctional sericin-nanohydroxyapatite/anthocyanidin microspheres *via* electrostatic injection. The incorporation of anthocyanidin endowed the microspheres with ROS scavenging ability and promoted the polarization of macrophages to the M2 phenotype. Furthermore, these microsphere scaffolds, capable of drug delivery, addressed the instability of anthocyanidin, providing a promising platform for the treatment of bone defects associated with chronic periodontitis [134].

#### Biomaterials based on electrical conductivity

**Figure 6.**
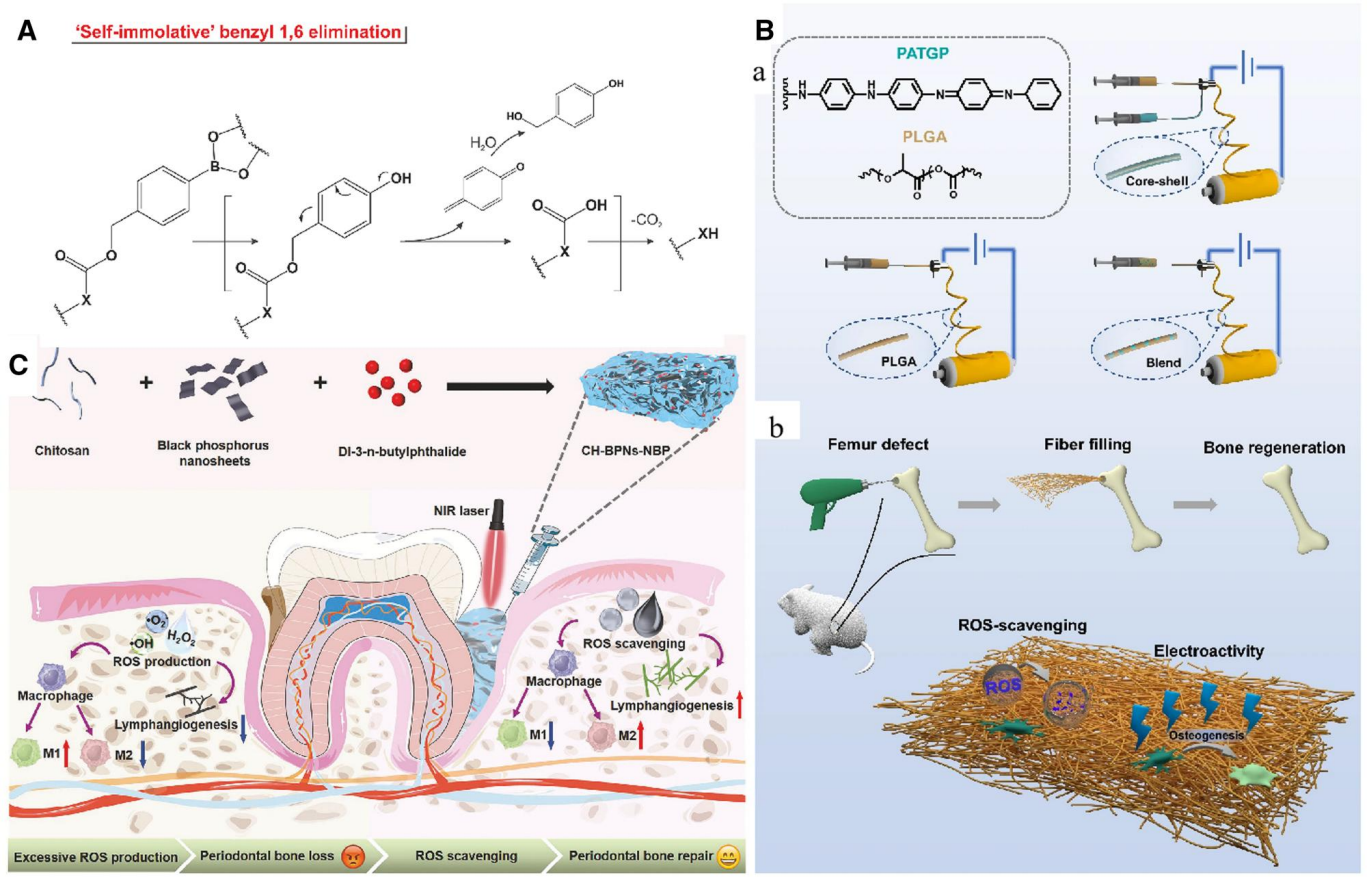
Representative biomaterials based on electrical conductivity and their application. (**A**) The free ROS scavenging mechanism of conductive materials. Conductive materials participate in redox reactions by donating or accepting electrons. Adapted with permission from Ref. [158]. Copyright 2019, Wiley-VCH Verlag. (**B**) Schematic illustration of the design and fabrication of nanofibers and their application in bone regeneration. PLGA/PATGP core-shell nanofibers performed the function of ROS-scavenging, electroactivity and osteogenesis. Adapted with permission from Ref. [136]. Copyright 2022, Donghua University, Shanghai, China (**C**) Schematic illustration of the synthesis of the CH-BPNs-NBP hydrogel and its application in treating periodontitis. BP nanosheets acted as ROS scavengers, while dl-3-n-butylphthalide promoted blood vessel formation. This dual action helps protect cells from oxidative stress and inflammation. Reproduced with permission from Ref. [138]. Copyright 2024, John Wiley and Sons Ltd.

Synthetic conductive biomaterials, primarily classified into organic and inorganic conductive materials, exhibit the potential for ROS scavenging (Figure 6). Among them, organic conductive materials typically contain groups with *π*-electron structure (e.g. aromatic rings) or structural units capable of donating free electrons (e.g. pyrrole, aniline, etc), such as polyaniline, polypyrrole, polythiophene, etc. [158]. Through the process of electron transfer, the benzene ring in organic macromolecule can donate free radical electrons, thereby stabilizing the material and reducing its activity. Huang *et al.* synthesized a kind of biodegradable conductive polyphosphazenes (PATGP) *via* reacting both the conductive aniline tetramer (AT) oligomer and glycine ethyl ester with poly(dichlorophosphazene) (PDCP). The AT endowed the PATGP with conductivity and antioxidant properties, which made them quite efficient in enhancing osteogenesis [135]. Furthermore, Huang *et al.* used co-axial electrospinning technique to fabricate PLGA/PATGP core-shell nanofibrous meshes. These blend nanofibers exhibited obvious electroactivity and presented capacity in scavenging ROS, reducing inflammation and enhancing osteogenesis [136].

The conductivity of inorganic conducting biomaterials is typically driven by the free electrons in metals, the electronic band structure of semiconductors or the conductive properties of carbon materials (e.g. *p* electrons in graphene) [159]. By reacting with free radicals, these conductive biomaterials can convert them into stable compounds *via* an electron transfer process. To enhance the electrical conductivity of certain non-metallic materials, which typically exhibit low conductivity, chemical modifications involving the incorporation of metallic or non-metallic elements are often required (Table 2) [160].

#### Silicon nitrogen oxide

The nitrogen-oxygen (N/O) ratio in the amorphous silicon oxynitride (SiONx) composition influences the localized surface dipole, which affects the oxidation or valence state of the surface. This variation in oxidation states leads to differential cellular responses, with cells exposed to the material’s surface in varying oxidation states exhibiting variations in viability, proliferation and differentiation. Ahuja *et al.* used SiONx as a coating for bone defect implants and found that increasing the N/O ratio enhanced the expression of cell-mediated bone markers in oxidative stress situations, as well as Nrf2 activity [137].

#### Black phosphorus

Black phosphorus (BP) is a nonmetallic material characterized by a multilayered structure, which can be converted into two-dimensional black phosphorus nanoparticles (BPN). The substantial specific surface area and the zero-valence state of phosphorus confer BPN with excellent ROS scavenging properties [138]. When combined with dl-3-n-butylphthalide (NBP), a vasodilator for the lymphatic vasculature of jaw vascular units, BPN showed cellular protection against ROS-induced damage and promoted macrophage M2 polarization as well as enhanced lymphatic function [138, 139]. These effects suggested a potential to stimulate osteogenesis and angiogenesis.

#### Graphene

Graphene is a single-atom-thick, two-dimensional sheet of hexagonally arranged carbon atoms [161]. Compared with traditional two-dimensional materials, two-dimensional transition metal carbides (MXenes) have better mechanical, magnetic and electrical properties [162]. The incorporation of inorganic MXene nanosheets into injectable scaffolds enhanced osteoconductivity, ROS scavenging and anti-inflammatory properties, as evidenced by their ability to support the regeneration of alveolar bone tissue in a rat model of periodontal disease [140]. Graphene quantum dots (GQDs) are a type of zero-dimensional carbon nanomaterial with a small lateral size, and they can be employed as an effective antioxidant due to the abundance of unpaired electrons and numerous, characteristics associated with reducing functional groups [163]. Therefore, GQDs often scavenge ROS by electron transfer and radical adduct formation [164]. Wu *et al.* significantly enhanced the material’s antioxidant capacity by doping GQD with nitrogen, thereby increasing their electron density [165]. Furthermore, GQDs could induce and enhance the osteogenic differentiation of PDLSCs by stimulating the Wnt/*β*-catenin signaling pathway in the inflammatory microenvironment [166].

#### Biomaterials based on reactive oxygen species-induced bond cleavage

**Figure 7.**
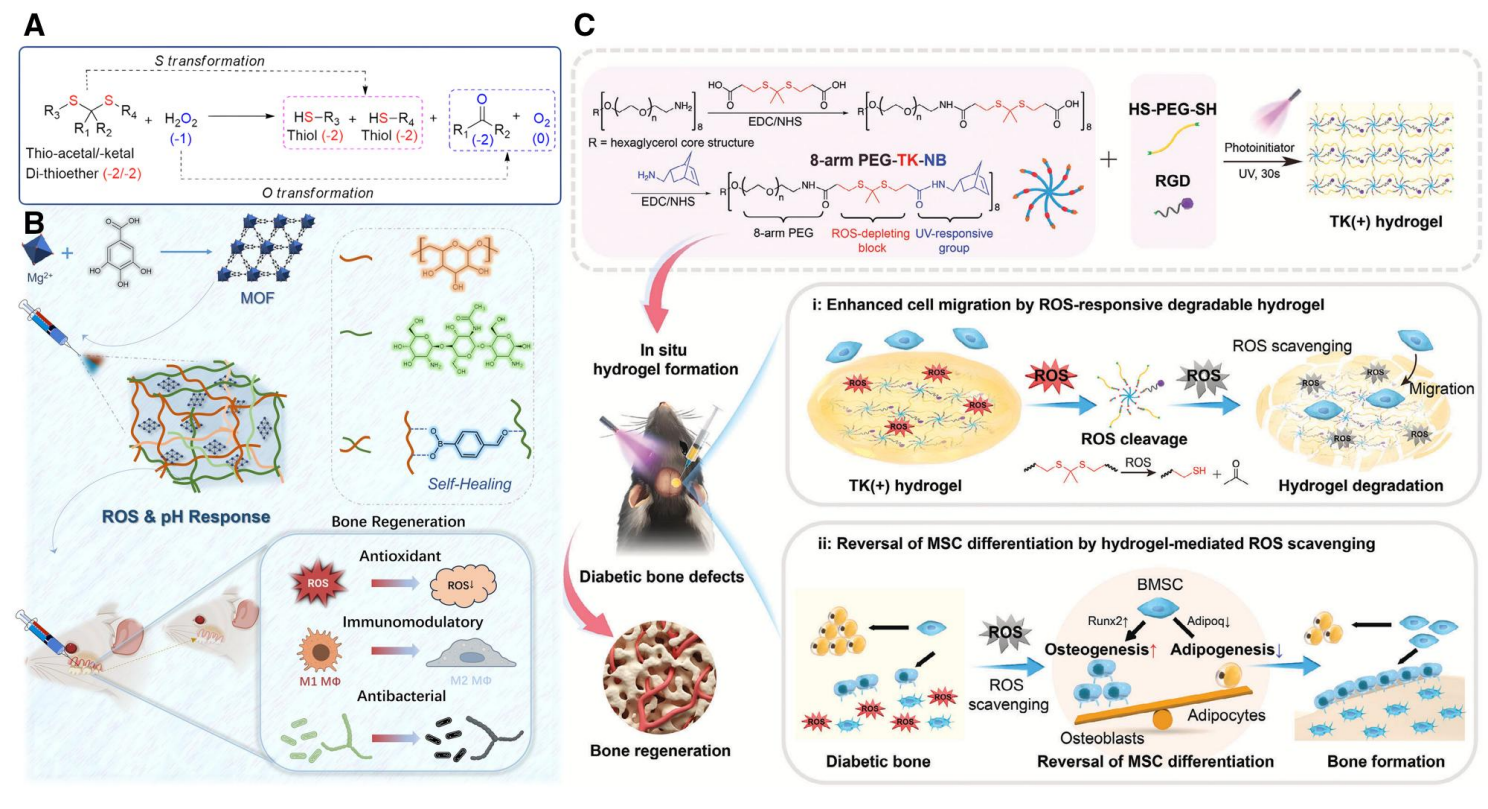
Representative biomaterials based on ROS-induced bond cleavage and their application. (**A**) The mechanism for the oxidative degradation of thioacetals/thioketals. Thioacetal and thioketal bonds undergo oxidative cleavage in the presence of ROS, resulting in the formation of thiols and aldehydes or ketones. Adapted with permission from Ref. [167]. Copyright 2020, Elsevier. (**B**) The schematic illustration of the preparation, application and bone regeneration promotion mechanism of CSBDX@MOF. The borate bond in the hydrogel crosslinked network exhibited ROS response and enabling on-demand drug release in response to high levels of ROS in the periodontitis environment. Reproduced with permission from Ref. [144]. Copyright 2024, BioMed Central. (**C**) Design and preparation of a ROS-responsive hydrogel with ROS scavenging and responsive degradation properties for diabetic bone regeneration. TK hydrogel exhibited the ability to scavenge ROS, enhance cell migration and reverse BMSC differentiation for promoting diabetic bone regeneration. Reproduced with permission from Ref. [147]. Copyright 2023 Wiley-VCH GmbH.

In recent years, integrating specific chemical bonds capable of effectively responding to ROS and triggering ROS scavenging is crucial. For the ROS-induced structural cleavage, ROS can react with various chemical groups such as phenylboronic acid, ester bonds and thioketals, hydrazone and disulfide linkages, leading to the structure cleavage and skeleton collapse. This process effectively removes ROS from the local environment, contributing to localized redox regulation, albeit in a noncatalytic, stoichiometric manner. Furthermore, by incorporating these ROS-responsive chemical bonds into drug delivery systems (like nanoparticles and hydrogels), therapeutic agents can be encapsulated and released specifically in response to elevated ROS levels (Figure 7; Table 2) [167–169].

#### Phenylboronic acid and its derivatives

Phenylboronic acid and its derivatives are compounds based on benzene containing a boron atom, with the basic structure consisting of a benzene ring bonded to one or more boronic acid groups (-B(OH)_2_). When reaction with H_2_O_2_ and ·OH, a phenylboronic acid typically undergoes oxidation, resulting in the formation of a phenylboronic acid oxidation product, wherein the carbon-boron bond (C-B) and the boron-oxygen bond (B-O) undergo changes [170]. The C-B bond can be cleaved, and electron transfer processes may occur in the phenylboronic acid molecule through interaction with superoxide anions [171]. The phenylboronic acid ester bond shows high sensitivity to H_2_O_2_, and bond breakage occurs within 24 h at a H_2_O_2_ concentration up to 50 μM [172]. Phenylboronic acid and its derivatives are among the materials synthesized for the construction of ROS-responsive frameworks. These frameworks often feature a common hydrogel network crosslinked with PVA, wherein chemical bonds are disrupted upon ROS oxidization, resulting in the release of the drug [141–143]. Luo *et al.* constructed a metal-organic framework (MOF) composed of magnesium (Mg) and gallic acid (GA), incorporating a smart responsive hydrogel system consisting of CMC, dextran (DEX) and 4-formylphenylboronic acid (4-FPBA). The borate bond in the hydrogel crosslinked network demonstrated ROS sensitivity, enabling on-demand drug release in response to high levels of ROS and providing favorable conditions for the improvement of bone regeneration microenvironment in periodontitis [144].

#### Thioketone

The chemical structure of thioketone (TK), a type of ketone compound in which the oxygen atom has been replaced by sulfur, typically features a carbon-sulfur double bond (C = S). This bond is highly electrophilic, allowing it to readily react with ROS, leading to the cleavage of the C = S bond [173]. Duvall *et al.* synthesized a poly(thioketone urethane) (PTKUR) biomaterial scaffold that was selectively degraded by ROS, due to the inherent ROS sensitivity of poly(thioketal) (PTK). This property showed promising applications for wound repair and bone regeneration [145, 146]. In addition, Zhang *et al.* developed a factor-free hydrogel that contained ROS-cleavable thioketal linkers and ultraviolet (UV)-responsive norbornene groups conjugated with 8-arm poly(ethylene glycol) macromers. ROS-cleavable thioketal linkers reduced high levels of ROS and reversed BMSC differentiation from adipogenic to osteogenic phenotype, leading to the effective repair of bone defects in diabetic mouse models [147].

## Conclusions

In recent years, the effects of ROS in oral bone regeneration have attracted increasing attention due to its dual role in physiological signaling and pathological injury. Starting from the pathological characteristics of oral bone metabolism, this review aims to clarify the relationship between the susceptible environment and ROS, and provides a detailed overview of the physiological homeostasis of ROS, as well as the mechanisms in which ROS contribute to oral bone formation and resorption. In addition, this review classifies ROS-regulating biomaterials according to the ROS scavenging mechanism and comprehensively summarizes the recent progress of ROS-regulating biomaterials in the field of oral bone regeneration. Existing ROS-regulating biomaterials have demonstrated some potential in scavenging ROS, reducing inflammation, promoting osteogenesis and enhancing tissue regeneration. However, the differentiation between physiological and pathological ROS, targeted regulation and clinical applicability still needs to be further improved. With the updated knowledge of oral bone biology and the continuous progress of material science, ROS regulation strategies will provide a solid foundation for realizing precise and effective treatment in the field of oral bone reconstruction.

## Challenges and perspectives

Biomaterials with the ability to regulate ROS, alleviate oxidative stress and induce tissue regeneration hold great promise for regenerative medicine and tissue regeneration [174, 175]. As described in the second part of this review, ROS plays a ‘double-edged sword’ role in oral bone formation and resorption, and its dynamic balance is the core of maintaining this homeostasis. However, pathological ROS imbalance drives bone resorption and inflammation, demonstrating the significance of precise regulation for repairing bone defects and blocking pathological processes. Currently, a variety of ROS-regulating biomaterials with different mechanisms have been developed for oral bone repair. However, there are still deficiencies in this field, and this section analyzes in detail the lack of the existing materials and proposes thoughts on future research directions.

### Spatiotemporal challenges of reactive oxygen species in balancing antibacterial activity and bone healing

**Figure 8.**
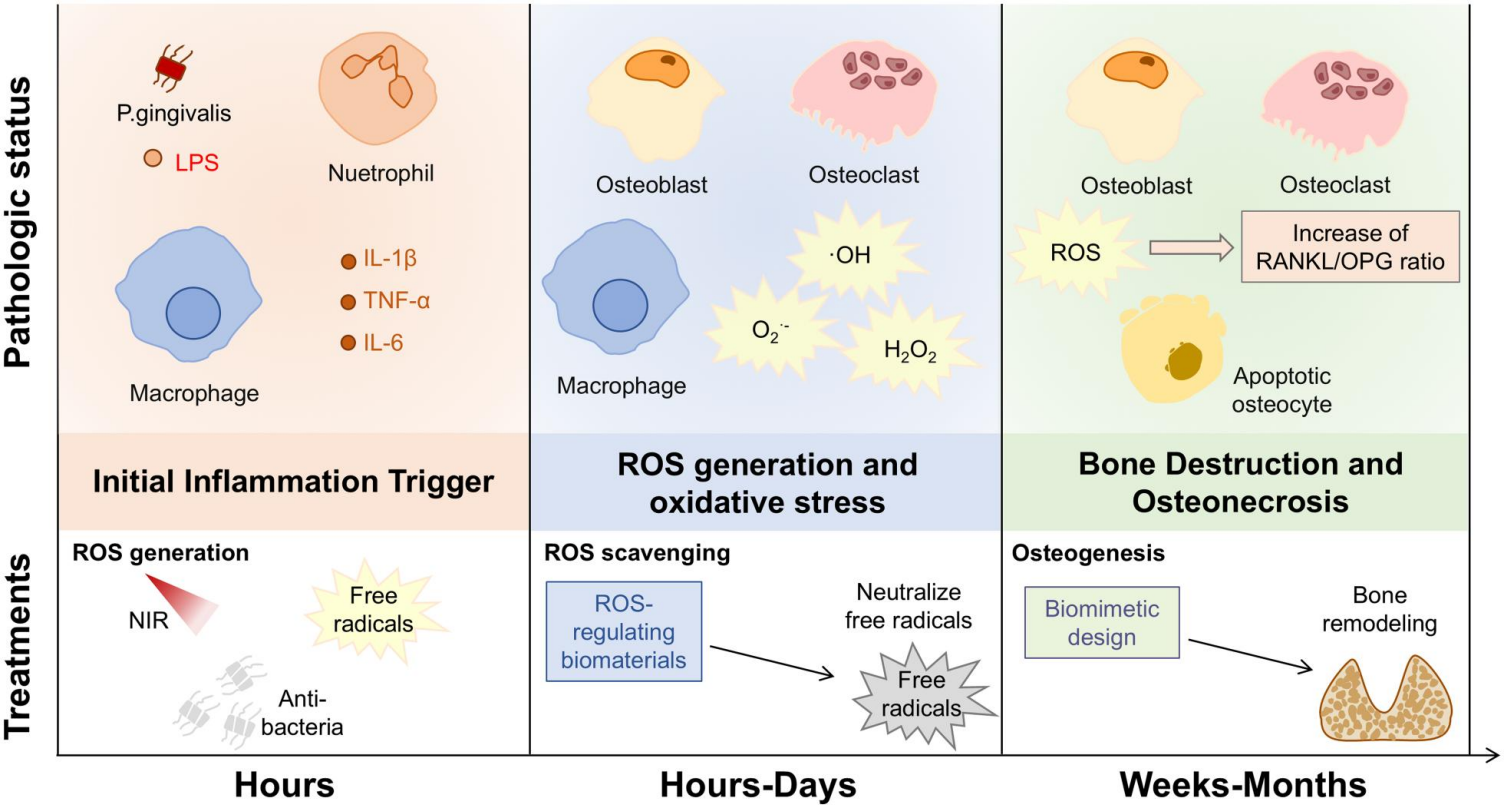
A timeline illustrated ROS/inflammation effect on oral bone. The ROS/inflammation effect involves an initial inflammatory trigger (left), leading to increased ROS production, oxidative stress (middle) and ultimately bone destruction (right). Several potential approaches target different stages of this process, including generating ROS to kill bacteria which is the main source of inflammation in oral bone, neutralizing ROS through applying ROS-regulating biomaterials and promoting bone regeneration by applying biomimetic design. LPS: Lipopolysaccharides. NIR: near-infrared spectroscopy.

ROS exhibit dual properties in the context of infectious oral bone defects. ROS play a crucial role in pathogen clearance during the early inflammatory process, but their prolonged or excessive presence may inhibit osteogenesis and exacerbate tissue damage. High ROS levels in infected areas during infection help clear bacteria, and the infectious phase usually lasts a few days [176]. However, during the period of bone repair and remodeling following the onset of infection, which usually lasts from weeks to months, bone regeneration requires low to moderate levels of ROS to support angiogenesis, OB activity and matrix remodeling (Figure 8) [177]. Therefore, bidirectional ROS-regulating is essential for maximizing therapy efficacy and mitigating the negative effects of diverse techniques in ROS-centered therapeutic approaches [178].

Currently, there are two feasible strategies could be considered: (1) Administering antioxidants following the action of ROS-generating agents or combining them directly with these agents. For example, ROS-generating therapies, including aPDT and sonodynamic therapy (SDT), have recently emerged as effective treatments for oral bacterial infections. These therapies use light and photoactivated sensitizers to generate ROS, which cause oxidative damage to bacteria at the site of infection without resistance concern [179]. Liu *et al.* designed a multi-functional composite UCNPs-Ce6-Mn(CO)_5_Br@Silane, which consists of several key components: Up-conversion nanoparticles (UCNPs: NaErF_4_: Tm^3+^@NaYF_4_: Yb^3+^), Ce6 and manganese pentacarbonyl bromide (Mn(CO)_5_Br). When exposed to near-infrared (NIR) light, the UCNPs emitted strong red light that triggered ROS generated by aPDT of Ce6. The generated ROS subsequently break the metal carbonyl bond of Mn(CO)_5_Br, leading to the production of carbon monoxide (CO) molecules and manganese ions (Mn^2+^), which further decompose H_2_O_2_ in the oxidative stress microenvironment [180]. (2) Modulating nanozyme activity *via* changing the microenvironment. Some nanozymes possess switchable enzyme-like activities that catalyze different reactions in different environments. For example, Lin *et al.* synthesized a piezoelectric hydrogel (ZeAHZ) containing zein, sodium alginate and heterojunction of high-entropy alloy and zinc sulfide that exhibits enhanced piezoelectricity and nanozyme activities. Under acidic circumstances and ultrasound, ZeAHZ’s piezoelectric effect enhanced peroxidase-like activity and sonodynamic efficiency, producing a large amount of ROS ($O_{2}^{\cdot -}$ and ·OH) to eliminate bacteria. On the other hand, due to the cascade reaction, ZeAHZ’s SOD-like activity and piezoelectric effect-enhanced CAT-like activity could scavenge ROS ($O_{2}^{\cdot -}$ and H_2_O_2_). Furthermore, ZeAHZ’s piezoelectric effect produced electrical stimulation that dramatically promotes OB proliferation and differentiation [181].

### Multithreshold design for selective reactive oxygen species responsiveness

The timing and extent of precise spatiotemporal regulation of ROS levels *in vivo* can profoundly influence outcomes. For instance, temporarily elevating ROS levels within 48 h following the creation of oral bone defects may help eliminate invasive bacteria, prevent infection and promote stem cell recruitment to the site [11, 182, 183]. However, sustained ROS elevation beyond one week disrupts redox homeostasis, potentially impairing extracellular matrix synthesis and mineralization of OBs, while also enhancing the activity of OCs, which could contribute to bone loss [18, 182, 184]. Significant challenge for ROS-regulating biomaterials lies in the design of materials capable of distinguishing between normal ROS concentrations and pathological ROS concentrations. The current development focus of most ROS-regulating biomaterials centers on the clearance of ROS, which may lead to excessive scavenging or ineffective intervention [40]. For example, NAC with broad-spectrum ROS scavenging may lead to excessive scavenging, inhibiting physiologic ROS required for osteogenesis and delaying bone formation [125]. Studies indicated that 10 μM H_2_O_2_-mediated preconditioning of BMSCs depicted an increased proliferation, while the addition of exogenous 125–150 μM H_2_O_2_ to BMSCs reduced activity of phosphatase, a marker of osteogenic differentiation [185, 186]. Nevertheless, the normal range of ROS levels in cells such as MSCs, OBs and OCs within the context of oral bone turnover has not been systematically studied [68, 186]. This gap in understanding presents a substantial challenge to the development of ROS-regulating biomaterials.

To address this challenge, research can be approached from the following directions: (1) Given the unique characteristics of oral bone and the lack of systematic studies on ROS levels, it is essential to encourage researchers to systematically investigate the ROS levels in oral bone, providing foundational data for the targeted design of ROS-regulating biomaterials under pathological conditions; (2) Achieving precise ROS regulation requires enhancing the sensitivity of ROS-regulating biomaterials to ROS stimuli. For example, phenylborate bonds are more reactive towards mild to moderate levels of H_2_O_2_, while disulfide bonds are more likely to be cleaved under high-intensity oxidative stress conditions [172, 187]. Furthermore, thioketals were cleaved selectively by high ROS concentration, particularly mM range of H_2_O_2_ concentration, while diselenide bonds were more sensitive to μM level of H_2_O_2_ for their lower energy [188, 189]. Although these study focused on accelerating oxidative reactions and thus destroying tumors, the composite strategy of these two materials not only allows for targeted drug delivery to ROS-prone areas but also realize the dynamic regulation of multi-threshold ROS [190].

### Phased, disease-specific strategies for reactive oxygen species regulation in oral bone defects

In fact, there are significant differences in the pathological mechanisms, microenvironmental characteristics and ROS dynamics of oral bone defects caused by different reasons. For example, bacterial plaque exacerbates ROS production, which contributes to the demineralization of tooth enamel and is a key process in dental caries; for inflammatory oral bone defects such as periodontitis, ROS production by immune cells during periodontal inflammation damages periodontal tissues and contributes to bone resorption [191, 192]. In addition, acute ROS burst exists in bone defects caused by trauma, which directly leads to cell membrane rupture and mitochondrial dysfunction, triggering the release of large quantities of $O_{2}^{\cdot -}$ and H_2_O_2_ and the formation of oxidative stress peaks [177]. Therefore, it is necessary to design targeted ROS-regulating strategies.

To address this challenge, research can be approached from the following directions: (1) Matching the different therapeutic effects of biomaterials to the progression of disease (e.g. from infection to healing) in a time-sequenced or condition-adapted manner. For example, time-programmed release systems that use biodegradable layers or hydrogel matrices with staggered degradation rates. Gao *et al*. designed a multilayered regenerated silk fibroin coating loaded with curcumin and Zn^2+^ on the surface of the polyethylene terephthalate grafts for providing time-programmed regulation. The materials initially released curcumin to inhibit the expression of inflammatory cytokines during the inflammatory phase, and released Zn^2+^ to induce the osteogenic differentiation of BMSCs during the regeneration phase [193]. (2) Disease-responsive smart materials that respond to local signals. For example, pH: acidic environment during infection triggers antimicrobial response; Enzymes such as matrix metalloproteinase (MMP): overexpression in chronic inflammation triggers therapeutic activation; ROS: material releases inclusions at high ROS levels in the early stages and stops releasing when redox homeostasis is restored. Hu *et al*. developed novel pH-activated nanoparticles synthesized from a quaternary ammonium chitosan. The nanoparticles achieved superb inhibition of free mixed bacteria and biofilm formation and prevention of alveolar bone absorption in periodontitis microenvironment [194]. (3) Multicompartment system (modular design): Different payloads are encapsulated in physically or chemically separated compartments within the material. Each compartment responds to a different stage of the disease or healing process. For example, core-shell nanoparticles: core for bone regeneration, shell for antimicrobial. Wu *et al*. developed a spatiotemporal drug-release PDA-functionalized mesoporous silicon nanoparticles core/shell drug delivery system loaded with osteogenic dexamethasone. Specifically, antibacterial silver nanoparticles were introduced into the PDA shell *via* a situ growth technique. This spatiotemporal drug-release system achieved antibiosis before effective osteogenesis caused by the release of osteogenic drugs [195]. (4) External trigger system: Combines ROS response with light, ultrasound or magnetic field response to control different phases of treatment. For example, external NIR light triggers ROS clearance after the initial antimicrobial phase is complete. Xiu *et al.* developed composite microspheres modified with a PDA layer, which exhibited the ability of ROS scavenging upon NIR irradiation [121].

### Challenges of clinical translation

ROS-regulating biomaterials have demonstrated significant promise in preclinical studies related to oral bone repair. Although there are few clinical studies in this field, they have shown significant preclinical transformation potential and gradually clear clinical application value in clinical studies. Currently, three registered clinical trials are in progress, as shown in Table 3. As described in section Biomaterials based on non-cleavage chemistry this review, quercetin exhibits antioxidant properties; however, its poor water solubility and low bioavailability limit its therapeutic potential. The clinical trial NCT05928546 investigates the use of a quercetin-loaded nanoemulsion as an adjunct to conventional periodontitis treatment. Additionally, the NCT02442453 clinical trial explores the impact of curcumin gel, in combination with scaling and root planing, on salivary oxidative stress levels in patients with chronic periodontitis, mainly by measuring SOD activity and the lipid peroxidation product malondialdehyde (MDA).

**Table 3.** Clinical trials of ROS-regulating strategies in oral bone repair

Active ingredients	Applications	Clinical Trials ·gov· Identifier	Phase	Recruitment status
Quercetin nanoemulgel	Periodontitis therapy	NCT05928546	Phase1	Not yet recruiting
Curcuma Longa Gel	Chronic periodontitis therapy	NCT02442453	Phase4	Completed
Tocopherol	Treatment of medication-related Osteonecrosis of the jaw (MRONJ)	NCT03040778	Phase3	Enrolling by invitation

Although the clinical translation of ROS-regulating biomaterials has begun to take shape, numerous challenges remain within this field. Notably, only three primary active ingredients involved in ROS regulation in clinical trials are all derived from natural plant extracts, with no clinical trials yet involving synthetic ROS-regulating biomaterials as the main components. This discrepancy may stem from the uncertainties surrounding the behavior of synthetic ROS-regulating biomaterials and their degradation products under both normal and pathological physiological conditions. Additionally, there is a lack of comprehensive toxicity assessments from *in vitro* and *in vivo* studies [196]. Given the current delay in the clinical translation of synthetically engineered ROS-regulating biomaterials, future research can consider the following aspects: (1) First, efforts should be made to mimic the structure and function of natural materials to reduce the uncertainties associated with the degradation products of synthetic biomaterials, while simultaneously enhancing the analysis of these degradation products. (2) A more comprehensive, multidimensional animal experimental framework should be established, including organoid toxicity testing, progression from small animal models to large animal models, thorough preclinical investigations and an analysis of the metabolic profiles of degradation products *in vivo*. These approaches will help lay a solid foundation for subsequent clinical trials.

### The reactive oxygen species regulation evaluation system needs to be improved

Last but not least, it is important to acknowledge that the current evaluation system for ROS regulation remains imperfect, and the detection methods employed in existing clinical trials fail to accurately capture the dynamic fluctuations of direct ROS levels. In clinical trials, the indirect assessment of oxidative stress levels serves as an easily observable and monitorable indicator. For example, in order to evaluate the effect of the active ingredient curcumin, the NCT02442453 clinical trial used indicators such as SOD levels and free radical damage in the form of MDA in the saliva of patients with chronic periodontitis to assess the presence of oxidative stress and explore the therapeutic effect of turmeric gel as a local ROS regulating ingredient.

In addition to the indirect measurement modalities, direct ROS assays are a valuable and promising biomarker that can reflect on disease status, which include fluorescence-based methods such as 2’,7’-dichlorodihydrofluorescein diacetate (DCFDA) and dihydroethidium (DHE), as well as electron paramagnetic resonance (EPR) spectroscopy like EPR spin-trapping and triarylmethyl free radical probes [197–199]. These essays provide direct evidence of ROS presence, allow for real-time measurements of ROS production, and may be more specific for certain ROS compared to indirect assays. However, their measurement in biological systems is a complex task given the short half-life and high reactivity of these species [197, 199]. For example, EPR measurements usually require low temperatures and long recording times for ROS detection in physiological conditions. The short lifetime and low concentration of free radicals *in vivo* detection by EPR are challenges [200]. Therefore, future advancements may involve the integration of direct ROS detection indicators, which more accurately reflect the dynamic changes in ROS levels, alongside indirect oxidative stress biomarkers, to provide a comprehensive evaluation of treatment efficacy.

## Supplementary Material

rbaf091_Supplementary_Data
