# Supplementary material for: Navigating oxidative stress in oral bone regeneration: mechanisms and reactive oxygen species-regulating biomaterial strategies
Source: Regen Biomater. 2025 Sep 1;12:rbaf091. doi: 10.1093/rb/rbaf091 (PMC12493052; doi:10.1093/rb/rbaf091)
Supplement: rbaf091_Supplementary_Data [file rbaf091_supplementary_data.zip › supplemmentary.docx]

**Supplementary materials**

**List of Abbreviations**

| Abbreviation | Full name | Abbreviation | Full name |
| --- | --- | --- | --- |
| ROS | Reactive oxygen species | X^·^ | Free radicals |
| NOX | Nicotinamide adenine dinucleotide phosphate hydrogen oxidase | EGCG | Epigallocatechin gallate |
| OB | Osteoblast | EA | Ellagic acid |
| MSC | Mesenchymal stem cell | PCL | Polycaprolactone |
| OC | Osteoclast | OSA-PBA | An injectable hydrogel with phenylboronic acid-functionalized oxidized sodium alginate |
| SOD | Superoxide dismutase | CMC | Carboxymethyl chitosan |
| GPx | Glutathione peroxidase | Mino | Minocycline |
| CeO_2_ | Cerium dioxide | MPN | Metal-phenolic network |
| Wnt | Wingless-related integration site | BMP2 | Bone morphogenetic protein 2 |
| SOST | Sclerostin | hPDLSC | Human periodontal stem cell |
| DKK1 | Dickkopf-related protein 1 | CGA | Chlorogenic acid |
| RANKL | Nuclear factor kappa-B ligand | LPS | Lipopolysaccharide |
| OPG | Osteoprotegerin | HGF | Human gingival fibroblast |
| RANK | Receptor activator of nuclear factor kappa-B | CysLT1R | Cysteinyl leukotriene receptor 1 |
| METC | Mitochondrial electron transport chain | Runx2 | Runt-related transcription factor 2 |
| O_2_^·-^ | Superoxide | BMSC | Bone marrow derived mesenchymal stem cell |
| H_2_O_2_ | Hydrogen peroxide | mTOR | Mammalian target of rapamycin |
| XOR | Xanthine oxidoreductase | ROO· | Peroxyl radicals |
| LOX | Lipoxygenase | DA | Dopamine |
| NADPH | Nicotinamide adenine dinucleotide phosphate hydrogen | PDA | Polydopamine |
| XO | Xanthine oxidase | SG | silk fibroin/gelatin |
| O_2_ | Oxygen molecule |  |  |
| H_2_O | Water molecule | PDA-mSF | Polydopamine-mediated ultralong silk microfiber |
| CAT | Catalase | Met | Metformin |
| MT | Metallothionein | ZIF | Zeolitic imidazolium framework |
| GSH | Glutathione | PGO | Polydopamine-mediated graphene oxide |
| -SH | Sulfhydryl groups | Se | Sericin |
| ·OH | Hydroxyl radical | PATGP | Polyphosphazene |
| NF-*κ*B | Nuclear factor kappa-B | AT | Aniline tetramer |
| TNF-*α* | Tumor necrosis factor receptor-alpha | PDCP | Poly(dichlorophosphazene) |
| TNFR1 | Tumor necrosis factor receptor-1 |  |  |
| m-BMSC | Jawbone-derived mesenchymal stem cell | N/O | Nitrogen-oxygen |
| t-BMSC | Tibia-derived mesenchymal stem cell | SiONx | Amorphous silicon oxynitride |
| PDLSC | Periodontal stem cell | BP | Black phosphorus |
| TGF-*β* | Transforming growth factor-*β* | BPN | Two-dimensional black phosphorus nanoparticles |
| PI3K | Phosphoinositide 3-kinase | NBP | dl-3-n-butylphthalide |
| AKT | Protein kinase B | MXene | Two-dimensional transition metal carbide |
| nM | Nanomolar | GQD | Graphene quantum dot |
| ATP | Adenosine triphosphate | -B(OH)_2_ | Boronic acid groups |
| ERK1/2 | Extracellular signal-regulated kinase | C-B | Carbon-boron bond |
| JNK | c-Jun N-terminal kinase | B-O | Boron-oxygen bond |
| MAPK | Mitogen-activated protein kinase | MOF | Metal-organic framework |
| FOXO | Forkhead box O | Mg | Magnesium |
| NAC | N-acetylcysteine | GA | Gallic acid |
| NLRP3 | Nucleotide-binding oligomerization domain-like receptor protein 3 | CMCS | Carboxymethyl chitosan |
| TREM2 | Triggering receptor expressed on myeloid cells-2 | DEX | Dextran |
| PLGA | Poly(D,L-lactide-co-glycolide) | 4-FPBA | 4-formylphenylboronic acid |
| GelMA | Gelatin methacrylate | TK | Thioketone |
| μM | Micromolar | C=S | Carbon-sulfur double bond |
| CoO | Cobalt oxide | PTKUR | Poly(thioketone urethane) |
| Ir | Iridium | PTK | Poly(thioketal) |
| Co | Cobalt | UV | ultraviolet |
| MVF | MIL-47(V)-F | SDT | Sonodynamic therapy |
| Nrf2 | Nuclear factor E2-related factor 2 | UCNP | Up-conversion nanoparticle |
| HO-1 | Heme oxygenase-1 | Mn(CO)_5_Br | Manganese pentacarbonyl bromide |
| Ce | Cerium | NIR | Near-infrared |
| HA@Ce-TA | A supramolecular network of cerium ion-coordinated tannic acid wrapped by nano-hydroxyapatite | CO | Carbon monoxide |
| TA | Tannic acid | Mn^2+^ | Manganese ion |
| Ce6 | Photosensitizer chlorin e6 | ZeAHZ | A piezoelectric hydrogel containing zein, sodium alginate, and heterojunction of high-entropy alloy and zinc sulfide |
| aPDT | Antimicrobial photodynamic therapy | MMP | matrix metalloproteinase |
| Mo | Molybdenum | MDA | Malondialdehyde |
| MnO_2_ | Manganese dioxide | DCFDA | 2’,7’-dichlorodihydrofluorescein diacetate |
| GBR | Guided bone regeneration | DHE | Dihydroethidium |
| PVA | Poly(vinyl alcohol) | EPR | Electron paramagnetic resonance |
| DOPA | 3,4-dihydroxy-D-phenylalanine | Steap4 | Six transmembrane epithelial antigen of the prostate 4 |
| ONOO^-^ | Peroxynitrite | Smad | Drosophila mothers against decapentaplegic protein |
| ClO^-^ | Hypochlorite | Cyt C | Cytochrome c |
| ROH | Polyphenol structure | FAK | [Focal adhesion kinase](https://pubmed.ncbi.nlm.nih.gov/37586468/) |
